# Abnormal Sialylation Promotes Chemotherapy Resistance in Bladder Cancer via the PI3K-AKT-mTOR Signaling Pathway

**DOI:** 10.3390/cancers18111713

**Published:** 2026-05-24

**Authors:** Junlong Zhu, Aimin Wang, Hang Tong, Yan Sun, Tinghao Li, Linfeng Wu, Xiaoyu Zhang, Zijia Qin, Weiyang He

**Affiliations:** Department of Urology, The First Affiliated Hospital of Chongqing Medical University, Chongqing 400016, China; 2023140143@hospital.cqmu.edu.cn (J.Z.); 2024110225@hospital.cqmu.edu.cn (A.W.); 204887@hospital.cqmu.edu.cn (H.T.); 2021130038@hospital.cqmu.edu.cn (Y.S.); 2022140056@hospital.cqmu.edu.cn (T.L.); 2025140195@hospital.cqmu.edu.cn (L.W.); 2022110221@hospital.cqmu.edu.cn (X.Z.); 2024140140@hospital.cqmu.edu.cn (Z.Q.)

**Keywords:** bladder cancer, microenvironment, gemcitabine–cisplatin resistance, sialylation, PI3K-AKT-mTOR signaling

## Abstract

Background: Aberrant glycosylation is closely associated with tumor progression, changes in the tumor microenvironment, and chemoresistance. This study aimed to identify prognostic sialylation-related genes in bladder cancer and define the role of ST3GAL6 in gemcitabine–cisplatin resistance. Methods: Molecular subtype analysis, prognostic analysis, and risk model construction were performed for sialylation-related genes using transcriptomic data and clinical information from the TCGA database. GC-resistant bladder cancer cell models were established for transcriptomic sequencing and untargeted metabolomic analysis. Cell proliferation and drug sensitivity assays were performed to evaluate the function of ST3GAL6. The regulatory relationship between IGF2BP3, ST3GAL6, and the PI3K pathway was further assessed by combining database analysis with molecular experiments. Results: Sialylation-related molecular patterns were associated with patient prognosis and tumor microenvironment features, particularly fibroblast-related characteristics, in bladder cancer. The key model gene ST3GAL6 was upregulated in bladder cancer tissues and was closely associated with prognosis. In GC-resistant bladder cancer cells, ST3GAL6 expression was significantly increased and accompanied by enhanced sialylation activity. ST3GAL6 promoted bladder cancer cell proliferation and reduced sensitivity to cisplatin and gemcitabine, at least in part through the PI3K-AKT-mTOR pathway. IGF2BP3 was also upregulated in resistant cells, is positively correlated with ST3GAL6, and may help maintain ST3GAL6’s expression by stabilizing its mRNA. Conclusions: Our findings suggest that aberrant sialylation is involved in bladder cancer progression and GC resistance. The IGF2BP3-ST3GAL6-PI3K/AKT/mTOR signaling axis may contribute to this process and may serve as a potential biomarker and therapeutic target in bladder cancer.

## Simple Summary

Bladder cancer is a common cancer of the urinary system, but patients often respond differently to treatment. Although gemcitabine plus cisplatin chemotherapy remains an important treatment option, some patients still experience recurrence, progression, or drug resistance. These differences may be influenced by changes within tumor cells and by the surrounding tumor microenvironment. This study focused on sialylation-related molecules, which are involved in sugar modifications on the cell surface, and explored their relationships with patient prognosis, tumor microenvironment features, and chemotherapy resistance in bladder cancer. The findings may help researchers better understand how bladder cancer progression and drug resistance develop. They may also provide useful clues for future risk assessment, prediction of treatment response, and development of new therapeutic strategies.

## 1. Introduction

Bladder cancer (BCa) is one of the most common malignancies in the urinary system. Due to its high incidence rate and mortality, it remains one of the most significant malignant tumors that threaten human health [1,2]. According to its muscle-layer invasion status, BCa is classified into non-muscle-invasive bladder cancer (NMIBC) and muscle-invasive bladder cancer (MIBC). NMIBC is mainly treated with local therapy and intravesical instillation therapy, but it has a high risk of recurrence and may progress to MIBC. MIBC is more aggressive and often requires radical cystectomy combined with cisplatin-based neoadjuvant or adjuvant chemotherapy [2]. However, some patients develop resistance during chemotherapy, which significantly affects their prognosis [3,4]. BCa is characterized by marked biological heterogeneity and complex mechanisms of drug resistance. As a result, the responses of patients with MIBC and advanced urothelial carcinoma to platinum-based chemotherapy, immunotherapy, and even newer combination regimens are often inconsistent [3,4,5]. Although immune checkpoint inhibitors, antibody–drug conjugates, and other emerging therapeutic strategies have brought new hope for advanced BCa, the overall prognosis of patients with advanced or metastatic disease remains unsatisfactory. Current treatment options still fail to fundamentally overcome recurrence, metastasis, and drug resistance [3,4]. Therefore, further elucidation of the molecular mechanisms underlying MIBC progression and therapeutic resistance at the basic research level, as well as the identification of novel therapeutic targets and biomarkers, is of important theoretical significance and potential clinical value for the treatment of MIBC [3,6].

Sialylation is a common terminal glycosylation modification of glycoproteins and glycolipids and plays an important role in regulating cell surface charge, molecular recognition, and signal transduction. Studies have shown that aberrant sialylation is an important molecular feature of multiple malignant tumors and contributes to tumor progression by promoting tumor cell adhesion, migration, invasion, metastasis, and immune evasion [7,8,9]. Hypersialylated glycans on the surface of tumor cells can bind to Siglec receptors expressed on immune cells, thereby activating inhibitory immune signaling and weakening antitumor immune responses. Therefore, the sialic acid–Siglec axis is considered a potential glyco-immune checkpoint associated with therapeutic resistance [10,11,12]. In addition, aberrant sialylation can alter tumor cell sensitivity to chemotherapy, immunotherapy, and other anticancer treatments by affecting membrane protein stability, receptor activation status, and downstream signaling pathways [7,13]. Although studies on sialylation in BCa remain limited, the existing evidence suggests that sialylation-related alterations are closely associated with BCa progression, prognosis, and treatment response and may represent an important component of the molecular heterogeneity of BCa [14,15,16,17]. Studies on the specific sialyltransferases, key substrates, and downstream molecular mechanisms involved, especially their roles in chemoresistance, remain insufficient. Therefore, further elucidation of the functions and mechanisms of sialylation in BCa may provide a new theoretical basis for biomarker discovery and the development of novel therapeutic targets for BCa.

N6-methyladenosine (m6A) is one of the most abundant endogenous post-transcriptional modifications of mRNA in mammals. It is dynamically regulated by methyltransferases, demethylases, and reader proteins and influences multiple aspects of RNA metabolism, including RNA stability, splicing, export, and translation. Aberrant m6A is closely associated not only with tumor cell proliferation, invasion, metabolic reprogramming, and remodeling of the tumor microenvironment but also with resistance to chemotherapy, targeted therapy, and immunotherapy [18,19,20]. In BCa, dysregulation of m6A is considered an important mechanism driving malignant progression and therapeutic resistance. Widespread aberrant m6A has been identified in muscle-invasive BCa. WTAP can promote malignant progression and suppress ferroptosis in BCa by targeting NRF2 in an m6A-dependent manner, whereas METTL16 can inhibit cisplatin resistance through an m6A-dependent autophagy pathway, suggesting that the m6A regulatory network plays a bidirectional role in the development of BCa chemoresistance [21,22,23]. There is also potential crosstalk between m6A and glycosylation. WTAP has been shown to mediate m6A of PIGT and enhance its mRNA stability, thereby promoting cell growth, glycolysis, and metastasis through regulation of GLUT1 glycosylation and membrane translocation [24]. However, studies on the coordinated regulation of BCa chemoresistance by m6A and glycosylation remain limited, and the key molecules involved and their downstream mechanisms have not yet been systematically elucidated. Therefore, further clarification of the functional interplay between these two processes will be significant for understanding the mechanisms of BCa chemoresistance and identifying new therapeutic targets.

Previous studies have shown that both aberrant sialylation and m6A modification are associated with tumor progression and therapeutic resistance. However, whether these two processes jointly contribute to chemoresistance in BCa, especially GC resistance, remains unclear. It is also not fully understood which sialylation-related genes are more closely associated with BCa prognosis and resistant phenotypes or whether these genes are regulated by m6A-related RNA binding proteins. Based on these questions, this study first analyzed the relationship between sialylation-related genes, molecular subtypes, and prognosis in the TCGA-BLCA cohort and constructed a prognostic risk model. We then combined GC-resistant cell models with transcriptomic and metabolomic data to further screen and validate the function of ST3GAL6. On this basis, we explored whether the m6A reader IGF2BP3 participates in GC resistance in BCa cells by affecting ST3GAL6 mRNA stability. By linking m6A-related post-transcriptional regulation, ST3GAL6-mediated sialylation changes, and GC resistance, this study aims to provide new experimental evidence for understanding the mechanisms of chemoresistance in BCa.

## 2. Materials and Methods

### 2.1. Cell Culture and Establishment of Drug-Resistant Cell Lines

Human BCa cell lines T24 (No. CL-0227) and UM-UC-3 (No. CL-0463) were purchased from Wuhan Procell Life Science & Technology Co., Ltd. (Wuhan, China) Cells were cultured in high-glucose DMEM supplemented with 10% fetal bovine serum and 1% penicillin–streptomycin and maintained in a cell incubator at 37 °C with 5% CO_2_.

To establish stable GC dual-resistant cell lines, the cells were continuously cultured in complete high-glucose DMEM containing cisplatin and gemcitabine. The initial concentrations of cisplatin and gemcitabine were 0.2 μg/mL and 5 ng/mL, respectively. When the cells adapted to the current drug concentrations and recovered stable proliferation, the drug concentrations were gradually increased. After approximately 10 months of stepwise selection, stable GC-resistant cell lines were obtained and designated T24/D-R and UM-UC-3/D-R. The final maintenance concentrations of cisplatin and gemcitabine were 1 μg/mL and 50 ng/mL, respectively.

### 2.2. Clinical Tissue Samples

A total of 5 pairs of BCa tissues and paired adjacent tissues confirmed by pathology after radical cystectomy in the Department of Urology, the First Affiliated Hospital of Chongqing Medical University, were collected. Tissue specimens used for immunohistochemistry were fixed in 4% paraformaldehyde, embedded in paraffin, and sectioned for subsequent staining. The remaining tissue specimens were stored at −80 °C. The clinical information of the patients from whom the tissue samples were obtained is provided in Table S1. This study was approved by the ethics committee of the First Affiliated Hospital of Chongqing Medical University, ethics batch number 2022-80, and all subjects signed the informed consent.

### 2.3. TCGA Data Acquisition and Sialylation-Related Gene Screening

The transcriptome expression data and corresponding clinical data of BCa were downloaded from TCGA. The expression matrix of 67 sialylation-related genes was extracted and combined with the clinical information. Univariate Cox regression analysis was used to screen candidate genes associated with prognosis, which were used for subsequent molecular typing and risk model construction.

### 2.4. Molecular Typing, Tumor Microenvironment Analysis, and Prognostic Model Construction

All bioinformatic analyses were performed in R 4.2.5, using the survival, NMF, caret, glmnet, survminer, timeROC, and ggpubr packages. Based on the expression profiles of sialylation-related genes and survival information from the TCGA-BLCA cohort, prognostic genes were first screened via univariate Cox regression analysis and then used for molecular subtyping with the NMF package. For NMF clustering, the number of clusters was set from 2 to 10, the Brunet algorithm was used, the analysis was repeated 10 times, and the random seed was set to 123456. According to the cophenetic coefficient and consensus matrix heatmap, the samples were finally divided into three subtypes: C1, C2, and C3. Survival differences among the subtypes were analyzed using the Kaplan–Meier method and log rank test, and the tumor microenvironment scores were merged with the subtype results by sample ID for comparison. For prognostic model construction, TCGA samples were randomly divided into training and validation sets at a 7:3 ratio using the caret package. In the training set, univariate Cox regression was first performed with a threshold of *p* < 0.05, followed by LASSO–Cox regression using the glmnet package. The candidate genes were then included in multivariate Cox regression to construct the risk score model. The risk score was calculated as follows: Risk score = Coef_ST3GAL5 × Exp_ST3GAL5 + Coef_ST3GAL6 × Exp_ST3GAL6 + Coef_SIGLEC6 × Exp_SIGLEC6 + Coef_SIGLEC10 × Exp_SIGLEC10 + Coef_B3GALT2 × Exp_B3GALT2. Coef represents the regression coefficient of the corresponding gene, and Exp represents its expression level. Patients were grouped using the median risk score as the cutoff, and the model performance was evaluated using the Kaplan–Meier survival analysis and the timeROC package.

### 2.5. Public Database Analysis and Functional Enrichment Analysis

Using the cbioportal database (https://www.cbioportal.org/) and the GEPIA 3 Database (https://gepia3.bioinfoliu.com/), the differences in prognosis and expression in this study were analyzed online.

### 2.6. Transcriptomic Sequencing and Metabolomic Analysis

UM-UC-3 and UM-UC-3/D-R cells in good growth condition and at the logarithmic growth phase were selected for transcriptome sequencing, with three samples included in each group. The total cellular RNA was extracted using the Trizol method, and the RNA concentration, purity, and integrity were assessed. RNA samples that passed quality control were sent to Tsingke Biotechnology Co., Ltd. (Beijing, China) for subsequent library construction and transcriptome sequencing. Quality control was performed on the sequencing data, followed by sequencing data analysis. Using UM-UC-3 cells as the control, differentially expressed genes in UM-UC-3/D-R cells were screened. The screening criteria for differentially expressed genes were |log2 fold change| > 1 and *p* < 0.05.

For untargeted metabolomic analysis, UM-UC-3 and UM-UC-3/D-R cells with stable growth and at the logarithmic growth phase were also selected, with six samples included in each group. Cells were washed with pre-cooled PBS and collected promptly, followed by metabolite extraction, according to the untargeted metabolomics workflow. The processed samples were sent to APExBIO (Houston, TX, USA) for detection using a liquid chromatography–tandem mass spectrometry (LC-MS/MS) platform. Quality control was performed on the mass spectrometry data, followed by data analysis. Differential metabolites were screened using the criteria of VIP > 1 and *p* < 0.05, and the fold change was also considered to evaluate the magnitude of metabolite changes.

### 2.7. shRNA and siRNA Transfection

ST3GAL6 knockdown lentivirus (pLKO.1-Puro vector), ST3GAL6 overexpression lentivirus (PCDH-CMV-MCS-EF1-Puro vector), and the corresponding control lentiviruses were constructed by Tsingke Biotechnology Co., Ltd. Before infection, cells were seeded into six-well plates and cultured until they reached approximately 40–60% confluence. The corresponding lentiviruses were then added for infection. After 24 h, the virus-containing medium was removed and replaced with fresh complete medium. At 48 h after infection, cells were selected with 1 μg/mL puromycin for 3–5 days to obtain stable transduced cell lines. After stable cell lines were established, the knockdown or overexpression efficiency of ST3GAL6 was verified via Western blot, and validated cells were used for subsequent experiments. The related shRNA and overexpression sequence information is listed in Table S4.

Transient knockdown of IGF2BP3 was performed using siRNA. The siRNA was synthesized by Shanghai GenePharma Co., Ltd. (Shanghai, China) Cells were seeded into six-well plates and transfected with siRNA when they reached approximately 50–70% confluence. The final siRNA concentration was 50 nM. Cells were collected 48 h after transfection for subsequent functional experiments and molecular analyses. The si-IGF2BP3 and negative control siRNA sequences are listed in Table S4.

### 2.8. RNA Extraction and Quantitative Real-Time PCR

Total cellular RNA was extracted using a rapid RNA extraction kit (Yishan Biotechnology, Shanghai, China, Cat. No. RN001-50Rxns), according to the manufacturer’s instructions. After RNA extraction, the RNA concentration and purity were measured. Total RNA was then reverse-transcribed into cDNA using an MCE reverse transcription kit (MCE, Cat. No. HY-K0501), and the cDNA was stored at −20 °C. Quantitative real-time PCR was performed using SYBR Green qPCR Mix (ABclonal, Wuhan, China, Cat. No. RK21204) on a real-time PCR system. β-actin was used as the internal reference gene, and the relative expression levels of target genes were calculated using the 2^−ΔΔCt^ method. All primer sequences are listed in Table S4.

### 2.9. Western Blot Analysis and Immunohistochemistry

The total cellular protein was extracted using RIPA lysis buffer (Epizyme, Cambridge, MA, USA, Cat. No. PC101). Equal amounts of protein samples were separated by electrophoresis and then transferred onto PVDF membranes (Beyotime, Shanghai, China, Cat. No. FFP22). Protein transfer was performed using an ice-bath-free rapid transfer buffer (Boster, Pleasanton, CA, USA, Cat. No. AR0050) at a constant current of 400 mA for 30–40 min. After transfer, the membranes were blocked with protein-free rapid blocking buffer (Boster, Cat. No. AR0041) at room temperature for approximately 20 min. The PVDF membranes were then incubated with the corresponding primary antibodies overnight at 4 °C. After primary antibody incubation, the membranes were washed three times with TBST for 5–10 min each, followed by incubation with the corresponding HRP-conjugated secondary antibodies at 25 °C for 1 h. After secondary antibody incubation, the membranes were washed again three times with TBST. Protein bands were detected using the Omni-ECL™ Femto Light Chemiluminescence Kit (Epizyme, Cat. No. SQ201L) and imaged using a chemiluminescence imaging system. Antibody dilutions ranged from 1:1000 to 1:5000, and the detailed manufacturers, catalog numbers, and dilution ratios of the antibodies are listed in Table S5.

Immunohistochemical staining was performed on paraffin-embedded sections. The sections were first treated with dewaxing solution and then rehydrated through a graded ethanol series to distilled water. Antigen retrieval was performed under appropriate conditions according to the requirements of each antibody, with care taken to avoid drying of the sections during this process. After natural cooling, the sections were washed with PBS. To reduce nonspecific background staining, the sections were incubated with 3% hydrogen peroxide in the dark for 25 min to block endogenous peroxidase activity, washed with PBS, and then blocked with 3% BSA at room temperature for 30 min. After removal of the blocking solution, the diluted primary antibody was added, and the sections were incubated overnight at 4 °C in a humidified chamber. The next day, after thorough washing with PBS, the sections were incubated with the corresponding HRP-conjugated secondary antibody at room temperature for 50 min. DAB was then used for color development, and the staining time was monitored under a microscope. Once a brown–yellow signal appeared, the reaction was stopped with water. The sections were then counterstained with hematoxylin, differentiated, blued, dehydrated, cleared, and mounted. The staining results were observed and photographed under a bright-field microscope. The corresponding antibody information is listed in Table S5.

### 2.10. Cell Proliferation Experiment

The cell proliferation was assessed using CCK-8 and EdU assays. For the CCK-8 assay, cells from each treatment group were seeded into 96-well plates (Corning, Corning, NY, USA, Cat. No. 3596) at a density of 1 × 10^4^ cells per well, with five replicate wells for each group. After cell attachment, the cells were cultured for an additional 48 h. For detection, the culture medium was carefully removed, and CCK-8 working solution (TargetMol, Wellesley Hills, MA, USA, Cat. No. C0005) was prepared at a concentration of 10%. A total of 100 μL of working solution was added to each well, and blank control wells were included. The plates were then incubated at 37 °C in the dark for 0.5–1 h. After incubation, the absorbance at 450 nm was measured using a microplate reader to evaluate cell proliferation.

The EdU assay was performed using an EdU cell proliferation detection kit (Beyotime, Cat. No. C0071S). Cells from different treatment groups were seeded into six-well plates (Corning, Cat. No. 3516). After cell attachment and once an appropriate cell density was reached, EdU working solution was added and incubated for 2 h. The cells were then fixed with 4% paraformaldehyde, permeabilized, subjected to EdU staining, and counterstained with DAPI. Images were captured under a fluorescence microscope, and the proportion of EdU-positive cells was calculated to reflect the level of cell proliferation.

### 2.11. Cell Cycle Detection

Cell cycle detection was performed using a Cell Cycle Detection Kit (Beyotime, Cat. No. C1052). Cells from each treatment group were collected, washed with pre-cooled PBS, and then fixed, treated with RNase A, and stained with PI, according to the manufacturer’s instructions. After staining, the cellular DNA content was measured using a CytoFLEX flow cytometer (Beckman Coulter, Brea, CA, USA). PI red fluorescence was acquired under 488 nm excitation, while forward scatter and side scatter signals were also recorded. At least 10,000 cell events were collected for each sample. The obtained data were analyzed using CytExpert software 2.4 to calculate the proportions of cells in the G0/G1, S, and G2/M phases.

### 2.12. Drug Sensitivity Detection and IC50 Calculation

The cells in different treatment groups were inoculated and treated with different doses of drugs according to the experimental arrangement, after the cells adhered to the wall. Then, the cell viability was detected using the CCK-8 method, and the IC50 values of each group were calculated. The drug concentrations used for cell treatment are listed in Table S6.

### 2.13. Subcutaneous Tumorigenesis Experiment in Nude Mice

The subcutaneous xenograft experiment was performed using 4–6-week-old male BALB/c nude mice, and all animals were maintained under SPF conditions. T24 cells were used for the animal experiment. Cells in the logarithmic growth phase were collected and resuspended in PBS. Each group included four nude mice. Each mouse was subcutaneously inoculated with 5 × 10^6^ cells in a total volume of 100 μL into the right dorsal flank region. After inoculation, the general condition of the mice and the tumor growth were monitored regularly: the tumor length and width were measured with calipers every 3 days, and the tumor volume was calculated using the formula V = 0.5 × length × width^2^. Humane endpoints were defined as achievement of the experimental objective, tumor diameter exceeding 20 mm, marked body weight loss of 15–20%, obvious moribund signs such as marked cyanosis of the lips or respiratory depression, extensive skin infection, or abnormal central nervous system responses such as convulsions, tremors, paralysis, or head tilt. When the experimental endpoint or humane endpoint was reached, the mice were euthanized by CO_2_ asphyxiation. Tumor tissues were then excised, photographed, weighed, and used for subsequent analyses.

### 2.14. Statistical Analysis

In vitro experiments were performed at least three independent times. Statistical analyses were performed using GraphPad Prism 8.0. Before parametric tests were applied, data normality was assessed using the Shapiro–Wilk test, and the homogeneity of variance was evaluated using Levene’s test. For data that met the assumptions of normality and equal variance, comparisons between two groups were performed using an unpaired two-tailed Student’s *t*-test, whereas comparisons among multiple groups were performed using one-way ANOVA. For data that did not meet the assumptions for parametric tests, appropriate non-parametric tests were used. Tumor growth curves were compared using two-way ANOVA. Survival curves were generated using the Kaplan–Meier method and compared using the log-rank test. A value of *p* < 0.05 was considered statistically significant.

## 3. Results

### 3.1. Sialylation Is Closely Associated with the Prognosis of BCa

**Figure 1:**
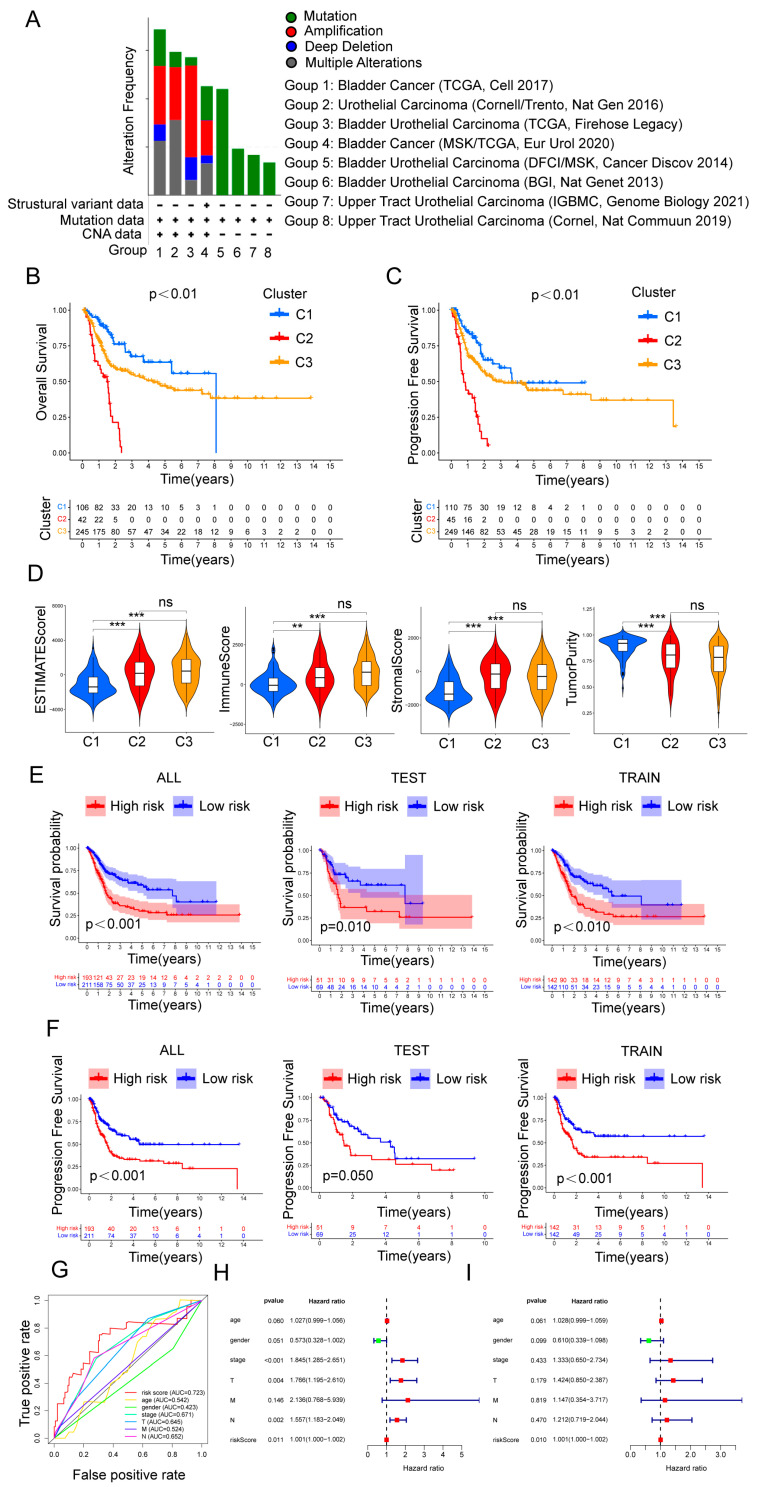
Sialylation is closely associated with the prognosis of BCa. (**A**) Genetic alterations in sialylation-related genes in BCa. (**B**,**C**) OS and PFS analyses of BCa patients after NMF clustering. (**D**) Tumor microenvironment-related scores in the C1, C2, and C3 subtypes. (**E**,**F**) OS and PFS analyses of the prognostic model. (**G**) Receiver operating characteristic (ROC) curves evaluating the predictive accuracy of the prognostic model (AUC values: risk score = 0.723, age = 0.542, gender = 0.423, stage = 0.671, T = 0.645, M = 0.524, N = 0.625). (**H**) Univariate regression analysis evaluating the prognostic value of the risk score. (**I**) Multivariate regression analysis evaluating the prognostic value of the risk score. ** *p* < 0.01, *** *p* < 0.001.

We screened 67 mRNAs involved in sialylation and extracted their expression data in patients with BCa from the TCGA, which were subsequently integrated with the corresponding clinical information (Table S2) [8,25,26,27,28,29]. Analysis using the cBioPortal database revealed that these 67 genes were significantly altered in BCa (Figure 1A). We then applied non-negative matrix factorization (NMF) for unsupervised clustering of prognosis-related genes and ultimately classified the samples into three molecular subtypes, designated C1, C2, and C3. The survival analysis demonstrated that patients in the C1 and C3 subtypes had significantly better overall survival (OS) and progression-free survival (PFS) than those in the C2 subtype (Figure 1B,C). Then, we evaluated the immune infiltration characteristics of the different molecular subtypes using the MCP-counter algorithm. The results showed that the tumor microenvironment composition of the C2 and C3 subtypes differed markedly from that of the C1 subtype. Compared with C1, the C2 and C3 subtypes exhibited significantly higher infiltration scores for CD8 T cells, cytotoxic lymphocytes, NK cells, monocytic lineage cells, and neutrophils. In addition, fibroblast infiltration was also significantly increased. These findings indicate that the C2 and C3 subtypes were characterized not only by higher levels of immune cell infiltration but also by more prominent stromal activation (Supplementary Figure S1A). Further comparison of the tumor microenvironment-related scores among the three subtypes showed that the overall scores were higher in C2 and C3 than in C1, suggesting higher involvement of both immune and stromal components and a more active tumor microenvironment in these two subtypes (Figure 1D). Taken together with the immune infiltration results, these data suggest that the C2 and C3 subtypes may exhibit a tumor microenvironment characterized by increased immune cell recruitment accompanied by enhanced stromal remodeling.

Next, we constructed a prognostic model using the TCGA BCa cohort. Prognosis-related genes were first screened in the training set by univariate Cox regression, followed by dimensionality reduction using LASSO–Cox regression, and a prognostic risk model was subsequently established using multivariate Cox proportional hazards regression. The model included a total of five genes: ST3GAL5, ST3GAL6, SIGLEC6, SIGLEC10, and B3GALT2. Based on the expression levels of each gene and their corresponding coefficients, we calculated the risk scores and classified patients into high-risk (H group) and low-risk (L group) categories (Table S3). Concurrently, we constructed training and validation sets using the entire cohort and verified the differences in gene expression between the H and L groups across these sets (Supplementary Figure S1B). The survival analysis revealed that, across different datasets, patients in the L group exhibited superior OS and PFS compared to those in the H group (Figure 1E,F). Finally, the model performance was evaluated using the area under the receiver operating characteristic curve (AUC), and the results indicated that the model demonstrated good predictive accuracy (Figure 1G; Supplementary Figure S1C). To determine whether the risk score had independent prognostic significance, age, gender, clinical stage, T stage, N stage, M stage, and risk score were included in the Cox regression analysis. Univariate analysis showed that stage, T stage, N stage, and risk score were associated with the prognosis of BCa patients. Further, multivariate analysis showed that the risk score remained statistically significant after adjustment for other clinicopathological factors, suggesting that this risk score may have potential independent prognostic value (Figure 1H,I). Through the prognostic model, we determined that sialylation is closely associated with the prognosis of BCA patients.

### 3.2. ST3GAL6 Can Affect the Proliferation of BCa Cells

**Figure 2:**
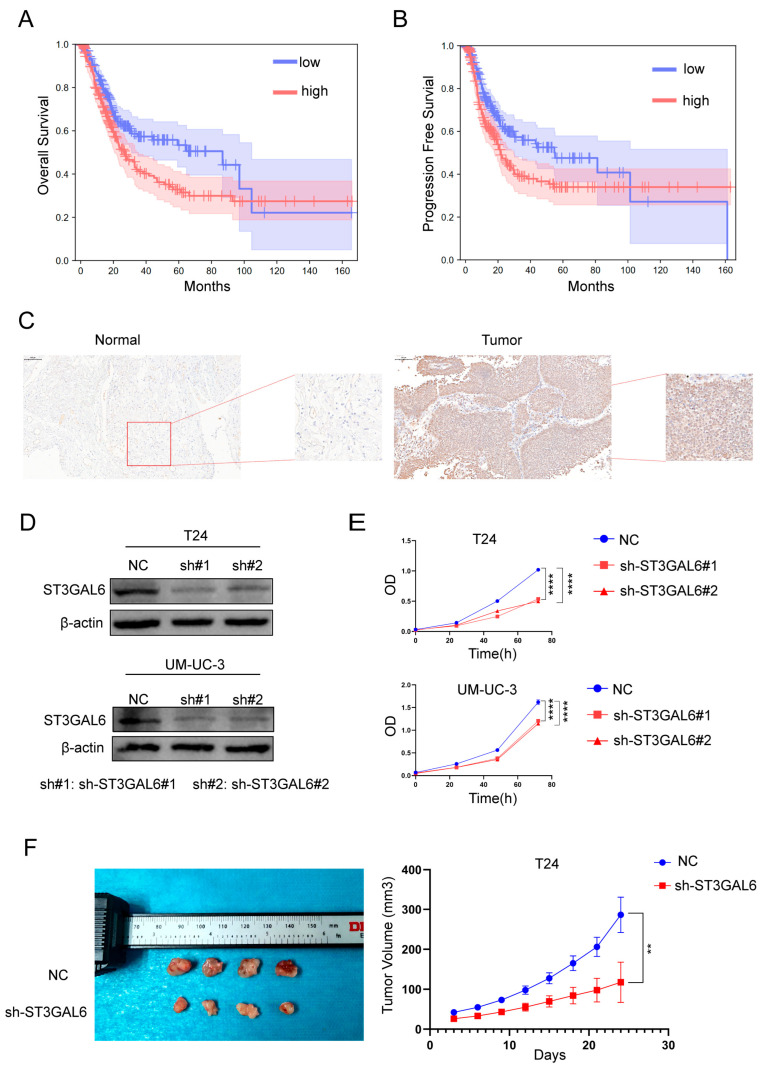
ST3GAL6 is highly expressed in BCa and promotes BCa cell proliferation. (**A**,**B**) OS and PFS analyses of patients with high and low ST3GAL6 expression. (**C**) Immunohistochemical analysis of differential ST3GAL6 expression in BCa tissues and adjacent normal tissues. (**D**) Knockdown efficiency of ST3GAL6. (**E**) ST3GAL6 regulates the proliferation of BCa cells in vitro. (**F**) ST3GAL6 promotes BCa progression in vivo. ** *p* < 0.01, **** *p* < 0.0001. The original Western blot figures can be found in Supplementary File S1.

We further performed prognostic analysis of the genes included in the above risk model using the GEPIA 3 database and determined that patients with high ST3GAL6 expression exhibited a significantly shorter median OS and PFS than those with low ST3GAL6 expression (Figure 2A,B; Table 1 and Table 2). Analysis of the collected paired BCa and adjacent normal tissues further showed that ST3GAL6 was upregulated in tumor tissues (Figure 2C). To further investigate the role of ST3GAL6 in BCa cells, we knocked down ST3GAL6 expression in T24 and UM-UC-3 cells (Figure 2D). Suppression of ST3GAL6 significantly inhibited the proliferation of both cell lines (Figure 2E; Supplementary Figure S2A). Flow cytometric analysis revealed that ST3GAL6 knockdown mainly induced S-phase arrest in BCa cells (Supplementary Figure S2B). In addition, the nude mouse xenograft experiments demonstrated that reduced ST3GAL6 expression impaired tumor growth in vivo and decreased the proliferative capacity of tumor tissues (Figure 2F). Taken together, these results indicate that ST3GAL6 plays an important role in promoting the proliferative process of BCa cells.

### 3.3. ST3GAL6 Affects Cisplatin and Gemcitabine Resistance in BCa Cells

**Figure 3:**
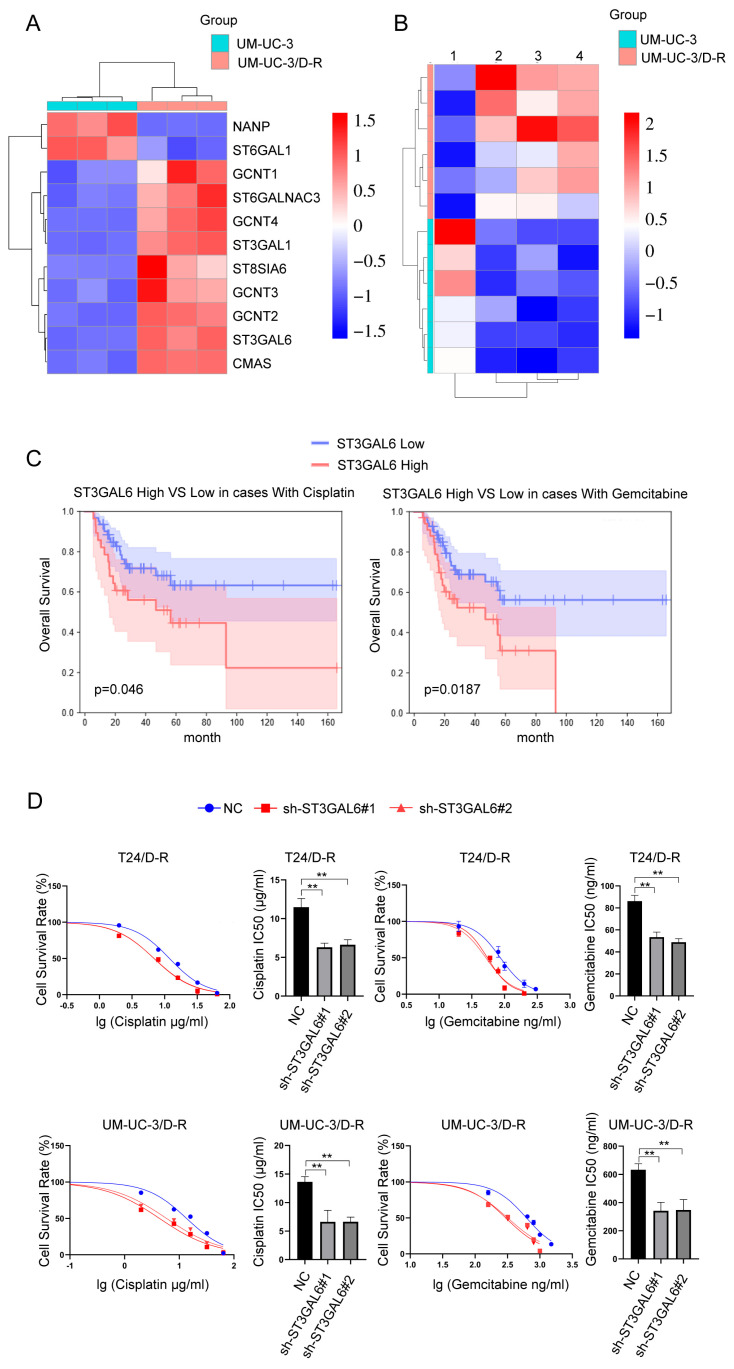
ST3GAL6 influences cisplatin and gemcitabine resistance in BCa cells. (**A**) Transcriptomic analysis of differential expression of sialylation-related genes in UM-UC-3 and UM-UC-3/D-R cells. (**B**) Metabolomic analysis of differential metabolites related to ST3GAL6 substrates (1: N-acetyl-D-lactosamine; 2: GM4; 3: CMP-N-acetylneuraminate; 4: N-acetylneuraminic acid). (**C**) OS analysis of patients with high and low ST3GAL6 expression after cisplatin treatment. (**D**) Effects of ST3GAL6 knockdown on the IC50 values of cisplatin and gemcitabine in BCa cells. ** *p* < 0.01.

At present, GC-based chemotherapy remains an important cornerstone treatment for patients with muscle-invasive BCa in the perioperative setting, as well as for some patients with advanced urothelial carcinoma. However, only a limited proportion of patients derive durable benefit from this regimen, and acquired resistance frequently develops during treatment, which markedly limits further improvement in therapeutic efficacy [3,30]. In this study, we established cisplatin- and gemcitabine-dual-resistant BCa cell lines, T24/D-R and UM-UC-3/D-R (Supplementary Figure S3A). Transcriptomic analysis of UM-UC-3 and UM-UC-3/D-R cells revealed that ST3GAL6 was significantly upregulated in UM-UC-3/D-R cells. Meanwhile, metabolomic analysis of UM-UC-3 and UM-UC-3/D-R cells showed a marked remodeling of sialic acid metabolism-related molecules in the resistant cells, characterized by significantly increased levels of N-acetylneuraminic acid and CMP-N-acetylneuraminate, decreased levels of the receptor-related glycan precursor N-acetyl-D-lactosamine, and increased levels of the sialylated glycolipid-related metabolite GM4, suggesting that the resistant cells possessed a higher sialylation potential under the drug-resistant state (Figure 3A,B). Analysis using the GEPIA 3 database further showed that patients with high ST3GAL6 expression had a shorter median OS after cisplatin or gemcitabine treatment than those with low ST3GAL6 expression (Figure 3C). In addition, no obvious differences in OS or PFS were observed after cisplatin treatment in patients with high ST3GAL6 expression, whereas cisplatin treatment was associated with a favorable prognosis in patients with low ST3GAL6 expression (Supplementary Figure S3B).

To determine whether ST3GAL6 is involved in the development of GC resistance in BCa, we established resistant cell lines with stable ST3GAL6 knockdown and parental cell lines with stable ST3GAL6 overexpression using lentiviral transduction (Supplementary Figure S3C,D). We found that downregulation of ST3GAL6 significantly reduced the IC50 values of cisplatin and gemcitabine in T24/D-R and UM-UC-3/D-R cells, whereas overexpression of ST3GAL6 increased the resistance of T24 and UM-UC-3 cells to both cisplatin and gemcitabine (Figure 3D, Supplementary Figure S3E). Collectively, these results suggest that ST3GAL6 is involved in the development of GC resistance in BCa cells.

### 3.4. ST3GAL6 Affects Cisplatin and Gemcitabine Resistance in BCa Cells Through the PI3K-AKT-mTOR Pathway

**Figure 4:**
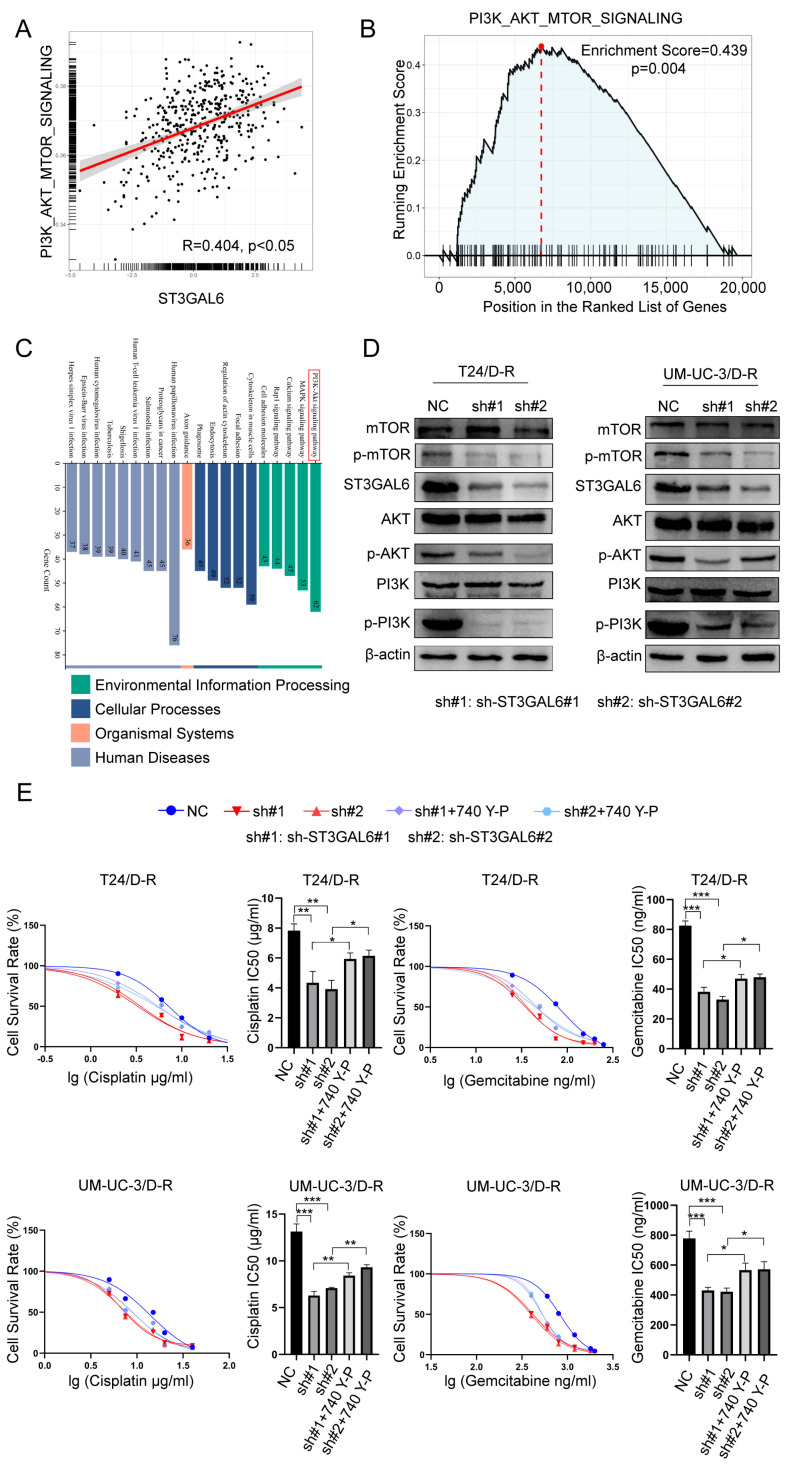
ST3GAL6 modulates cisplatin and gemcitabine resistance in BCa cells through the PI3K-AKT-mTOR pathway. (**A**,**B**) Enrichment of ST3GAL6 in the PI3K-AKT-mTOR pathway. (**C**) Enrichment of upregulated genes in UM-UC-3/D-R cells in the PI3K-AKT-mTOR pathway. (**D**) Downregulation of ST3GAL6 suppresses PI3K-AKT-mTOR pathway activity in resistant BCa cells. (**E**) Activation of the PI3K-AKT-mTOR pathway partially rescues the decrease in cisplatin and gemcitabine IC50 values caused by ST3GAL6 downregulation. * *p* < 0.05, ** *p* < 0.01, *** *p* < 0.001. The original Western blot figures can be found in Supplementary File S1.

To identify the potential signaling pathway through which ST3GAL6 exerts its function, we performed single-gene KEGG enrichment analysis of ST3GAL6 using the TCGA and determined that it was highly enriched in the PI3K-AKT-mTOR pathway (Figure 4A,B). Meanwhile, KEGG enrichment analysis of the upregulated genes in UM-UC-3-resistant cells also showed significant enrichment in the PI3K-AKT-mTOR pathway (Figure 4C). Based on these findings, we hypothesize that ST3GAL6 may regulate drug resistance in BCa cells by mediating the PI3K-AKT-mTOR pathway. In the resistant cells, we found that downregulation of ST3GAL6 reduced the activity of the PI3K-AKT-mTOR pathway (Figure 4D). After treatment of the established sh-ST3GAL6 stable cell lines with the PI3K agonist 740 Y-P, we observed that PI3K activation reversed the decrease in IC50 induced by ST3GAL6 knockdown in drug-resistant BCa cells (Figure 4E). These results suggest that ST3GAL6 may influence cisplatin and gemcitabine resistance in BCa cells through the PI3K-AKT-mTOR pathway.

### 3.5. IGF2BP3 Regulates BCa Chemoresistance Through the ST3GAL6-Mediated PI3K-AKT-mTOR Pathway

**Figure 5:**
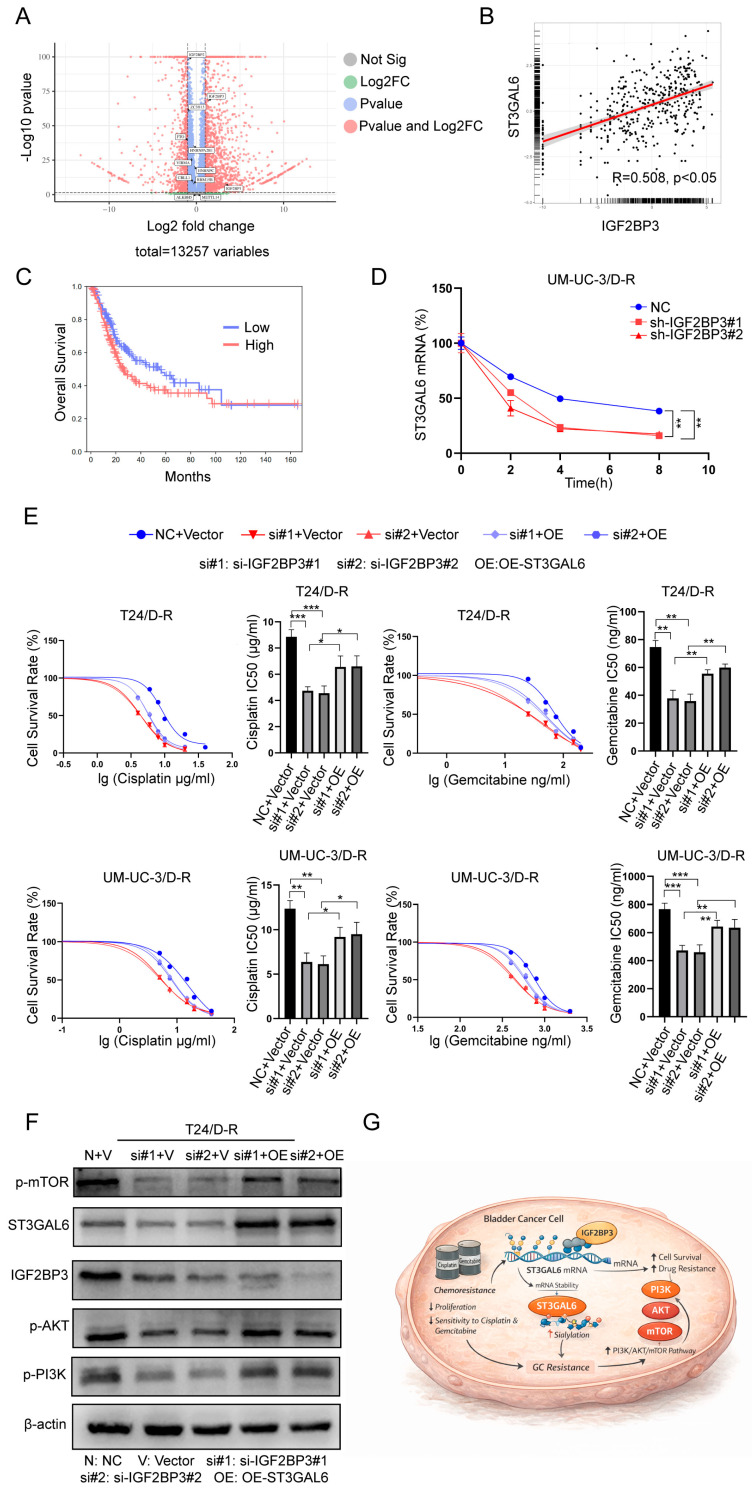
IGF2BP3 regulates BCa chemoresistance through ST3GAL6-mediated PI3K-AKT-mTOR signaling. (**A**) Differential expression analysis of m6A-related genes in UM-UC-3/D-R cells. (**B**) Correlation analysis between ST3GAL6 and IGF2BP3. (**C**) Overall OS analysis of patients with high and low IGF2BP3 expression. (**D**) Downregulation of IGF2BP3 reduces the stability of ST3GAL6 mRNA. (**E**) Downregulation of IGF2BP3 decreases the IC50 values of cisplatin and gemcitabine in resistant BCa cells, whereas ST3GAL6 overexpression partially rescues this reduction. (**F**) ST3GAL6 overexpression reverses the suppression of PI3K-AKT-mTOR pathway activity caused by IGF2BP3 depletion. (**G**) Schematic illustration of the proposed mechanism. * *p* < 0.05, ** *p* < 0.01, *** *p* < 0.001. The original Western blot figures can be found in Supplementary File S1.

Based on the previous transcriptomic data from UM-UC-3/D-R cells, we determined that ST3GAL6 mRNA was upregulated. As one of the most common RNA modifications, m6A plays an important role in regulating mRNA fate and can promote mRNA stability and translation. Therefore, we analyzed the expression of m6A-related genes in UM-UC-3/D-R cells and determined that only IGF2BP1 and IGF2BP3 were upregulated in the resistant cells (Figure 5A). Pearson correlation analysis further showed that IGF2BP3 was highly correlated with ST3GAL6, leading us to hypothesize that IGF2BP3 may serve as an upstream regulator of ST3GAL6 mRNA levels (Figure 5B; Supplementary Figure S4A).

Immunohistochemical analysis showed that IGF2BP3 was highly expressed in BCa tissues (Supplementary Figure S4B). We further assessed the association between IGF2BP3 expression and the prognosis of patients with BCa using the GEPIA 3 database and determined that patients with high IGF2BP3 expression had a shorter median OS than those with low expression (Figure 5C, Table 3). In patients with low IGF2BP3 expression, cisplatin treatment was associated with significantly improved OS and PFS compared with the untreated group, whereas no obvious differences in OS or PFS after cisplatin treatment were observed in the high-expression group (Supplementary Figure S4C).

KEGG enrichment analysis based on TCGA expression data further showed that IGF2BP3 was highly enriched in the PI3K-AKT-mTOR pathway across multiple tumor types (Supplementary Figure S4D–F). Combined with our previous findings, we reasonably speculated that IGF2BP3 may influence BCa chemoresistance through regulation of ST3GAL6 and subsequent activation of the PI3K-AKT-mTOR pathway. After knocking down IGF2BP3 in T24/D-R and UM-UC-3/D-R cells, we observed that the IC50 values of both cisplatin and gemcitabine were significantly reduced in the resistant cells. Notably, overexpression of ST3GAL6 rescued the decrease in IC50 caused by IGF2BP3 knockdown (Figure 5E). Following the treatment of the resistant cells with actinomycin D, we determined that the knockdown of IGF2BP3 reduced the stability of ST3GAL6 mRNA (Figure 5D). Meanwhile, downregulation of IGF2BP3 suppressed the activity of the PI3K-AKT-mTOR pathway, whereas increased ST3GAL6 expression was able to reverse the effects induced by IGF2BP3 depletion (Figure 5F, Supplementary Figure S4G). Taken together, these findings identify a novel IGF2BP3-ST3GAL6-PI3K-AKT-mTOR regulatory axis involved in cisplatin and gemcitabine resistance in BCa (Figure 5G).

## 4. Discussion

At present, GC-based chemotherapy remains an important cornerstone treatment for muscle-invasive BCa as well as locally advanced and metastatic urothelial carcinoma, playing a central role in both perioperative management and the systemic treatment of advanced disease [31]. However, the clinical benefit of GC therapy remains limited. Some patients are unable to tolerate standard cisplatin-based treatment because of impaired renal function, poor performance status, or comorbidities. Further, even among patients who are eligible for GC therapy, treatment responses vary substantially, and a considerable proportion of patients still experience recurrence, progression, or acquired resistance after treatment. Current clinical evaluation systems remain insufficient to accurately identify the subset of patients who can derive a durable benefit from GC therapy [30]. As systemic treatment for BCa gradually moves toward a more precise and individualized approach, current research has shifted from simply optimizing chemotherapy regimens to identifying molecular biomarkers that can reflect prognostic differences, predict drug sensitivity, and guide personalized therapeutic decision-making [32]. Therefore, in the context of GC treatment, the construction of a robust prognostic risk model through the integration of transcriptomic data and clinical information may not only facilitate more refined molecular risk stratification of patients with BCa but also provide clues for identifying key molecules associated with drug resistance, disease progression, and alterations in the tumor microenvironment. Based on this rationale, we first focused on sialylation-related genes, combined TCGA for molecular subtyping and risk model construction, and demonstrated significant differences in OS, PFS, and tumor microenvironment features among different subtypes and between high- and low-risk groups. These findings suggest that dysregulation of sialylation-related molecules may contribute to the heterogeneity of prognosis and treatment response in BCa and also provide a foundation for the subsequent identification of key functional genes and mechanistic investigation.

Aberrant sialylation is an important feature of glycosylation reprogramming in cancer. Sialic acids are usually located at the terminal ends of glycoprotein and glycolipid glycans and can influence cell-surface charge, glycocalyx structure, cell adhesion, receptor activation, and interactions between cells and the microenvironment. A highly sialylated state in tumor cells can promote resistance to apoptosis, proliferation, migration, invasion, and metastasis, and it is associated with poor prognosis [14,33]. In addition, sialylated glycans can bind to Siglec receptors on immune cells and induce immunosuppressive signaling. Recent studies have shown that sialylated glycoproteins on the surface of tumor cells can suppress immune cell cytotoxicity by binding to Siglec-7 and Siglec-9, suggesting that sialylation may act as a glyco-immune checkpoint involved in tumor immune escape [34]. ST3GAL6 belongs to the ST3GAL family and mainly catalyzes α2,3-linked sialylation. Previous studies have shown that ST3GAL6 is associated with the tumor stage, grade, and patient survival in urothelial bladder cancer, and ST3GAL6 knockdown reduces MAL-II-recognized α2,3-sialylation levels and suppresses the migration and invasion of BCa cells [35]. In addition, the ST3GAL6-AS1/ST3GAL6 axis has been reported to participate in colorectal cancer progression by regulating α2,3-sialylation and PI3K/Akt signaling [36]; ST3GAL6 has also been implicated in the regulation of hepatocellular carcinoma cell growth, migration, and invasion [37]. All these findings suggest that ST3GAL6-mediated aberrant sialylation may regulate malignant progression in multiple tumor types. In the TCGA, we determined that sialylation-related genes could stratify BCa patients into molecular subtypes with significantly different prognoses, and these subtypes exhibited marked differences in immune cell infiltration and tumor microenvironment scores. These findings suggest that aberrant sialylation is associated not only with patient survival outcomes but may also participate in tumor microenvironment remodeling and the development of heterogeneity in therapeutic response. Furthermore, ST3GAL6 was identified from our prognostic model and was confirmed to be upregulated in both BCa tissues and drug-resistant cells. Downregulation of ST3GAL6 simultaneously suppressed cell proliferation and reduced the IC50 values of cisplatin and gemcitabine. Combined with the metabolomic findings showing increased levels of N-acetylneuraminic acid and CMP-N-acetylneuraminate, together with decreased N-acetyl-D-lactosamine, these results indicate that BCa cells in the resistant state may possess a more active sialic acid metabolic flux and higher terminal sialylation potential, thereby providing a molecular basis for the maintenance of malignant phenotypes and drug resistance. Sialylation-related molecules often do not function in isolation but rather cooperate with canonical pro-survival signaling pathways to amplify malignant biological effects. In our study, ST3GAL6 was found to be highly associated with the PI3K-AKT-mTOR pathway, and activation of PI3K reversed the reduction in drug resistance caused by ST3GAL6 downregulation. This is consistent with previous reports showing that ST3GAL6/α2,3-sialylation can promote tumor progression through PI3K/AKT-related signaling. Therefore, we speculate that aberrant sialylation is unlikely to be a mere bystander in gemcitabine–cisplatin resistance in BCa; instead, it may serve as a critical node linking post-transcriptional regulation, metabolic remodeling, and activation of canonical survival signaling. Further investigation of ST3GAL6 and its downstream pathways will not only help elucidate the molecular basis underlying drug resistance in BCa but also provide a rationale for identifying prognostic biomarkers and potential therapeutic targets from glycosylation-related pathways.

As one of the most prevalent post-transcriptional epitranscriptomic modifications in eukaryotic cells, m6A has been demonstrated to be broadly involved in tumor cell proliferation, invasion, metastasis, and therapeutic resistance through the regulation of mRNA splicing, export, translation efficiency, and stability. Among these mechanisms, aberrant post-transcriptional regulation caused by imbalance of the “writers,” “erasers,” and “readers” has been recognized as an important driver of tumor progression and the evolution of drug resistance [38,39]. During the development of drug resistance, m6A does not simply affect the expression of a single gene; it more likely enables tumor cells to acquire sustained adaptability under therapeutic stress by stabilizing a series of transcripts associated with pro-survival signaling, proliferation, and metabolic reprogramming. For example, IGF2BP3, a canonical m6A reader, can recognize m6A-modified target mRNAs and delay their degradation, thereby enhancing the stability of oncogenic transcripts. Its high expression is generally closely associated with malignant progression and poor prognosis in cancer [40].

In BCa, IGF2BP3 has been shown to stabilize downstream target genes in an m6A-dependent manner and thereby promote cisplatin resistance. On the one hand, IGF2BP3 enhances BCa cell proliferation and cisplatin tolerance by increasing the stability of CDK6 mRNA [41]. On the other hand, IGF2BP3-mediated m6A regulation of cBio3 likewise contributes to the maintenance of the cisplatin-resistant phenotype in BCa cells [42]. In addition, the IGF2BP3–HMGB1 axis has also been demonstrated to be closely associated with BCa progression [43]. These findings suggest that the role of IGF2BP3 in BCa is not limited to that of a simple prognostic marker, but it may instead function as an important node linking aberrant m6A to therapeutic resistance. In our study, IGF2BP3 was significantly upregulated in the UM-UC-3/D-R-resistant cell line, and its expression showed a strong positive correlation with ST3GAL6. Actinomycin D assays demonstrated that knockdown of IGF2BP3 reduced the stability of ST3GAL6 mRNA, whereas overexpression of ST3GAL6 could partially rescue the drug-resistant phenotype and the suppression of the PI3K-AKT-mTOR pathway caused by IGF2BP3 downregulation. These results suggest that aberrant m6A may act upstream through IGF2BP3-mediated regulation of mRNA stability to sustain the high expression of the sialyltransferase ST3GAL6, thereby linking epitranscriptomic dysregulation to glycosylation reprogramming. Although reports directly showing that m6A regulates sialyltransferases to mediate drug resistance remain limited, the accumulating evidence has demonstrated that m6A broadly participates in tumor metabolic reprogramming and the regulation of canonical growth signaling pathways [42,44], while the PI3K/AKT pathway is itself one of the major downstream hubs through which m6A dysregulation influences tumor progression and therapeutic resistance [43]. Therefore, we propose that the IGF2BP3–ST3GAL6–PI3K-AKT-mTOR axis may represent a novel regulatory cascade in gemcitabine–cisplatin resistance in BCa, namely an “m6A dysregulation–aberrant sialylation–pro-survival signaling activation” axis. In this model, upstream IGF2BP3 sustains high ST3GAL6 expression by enhancing the stability of its mRNA, which in turn amplifies the PI3K-AKT-mTOR signaling activity, and ultimately promotes tumor cell proliferation, as well as resistance to cisplatin and gemcitabine.

The PI3K-AKT-mTOR pathway plays a central role in regulating cell proliferation, suppression of apoptosis, metabolic reprogramming, autophagy, epithelial–mesenchymal transition, and adaptation to the tumor microenvironment. Aberrant activation of this pathway is not only an important molecular basis for the sustained growth, invasion, and metastasis in multiple malignancies but is also closely associated with acquired drug resistance. Upstream, this pathway can be driven by receptor tyrosine kinases, PIK3CA mutations, PTEN loss, and various epigenetic regulatory events. Downstream, it promotes protein synthesis, maintains metabolic homeostasis, and suppresses cell death programs, thereby helping tumor cells acquire a sustained survival advantage under therapeutic stress [45,46,47]. Importantly, the PI3K-AKT-mTOR pathway should not be viewed merely as a concomitantly activated signaling cascade during the development of resistance; rather, it may function as an integrative hub that connects DNA damage responses, anti-apoptotic signaling, stemness maintenance, and metabolic adaptation, thereby attenuating the cytotoxic effects of chemotherapy, targeted therapy, and even immunotherapy [42,43]. In BCa, aberrant activation of this pathway has likewise been considered closely related to enhanced tumor cell proliferation, migration, invasion, and reduced chemosensitivity. m6A-related molecules can influence cisplatin sensitivity in BCa cells through the PI3K-AKT-mTOR axis, while molecules such as PRDX2 can regulate tumor stemness, the EMT phenotype, and cisplatin resistance by modulating PI3K-AKT and mTOR signaling [48,49,50].

To address the clinical challenge whereby the benefit of GC therapy in BCa remains limited, while recurrence, progression, and acquired resistance are common, we first performed a systematic integrative analysis of sialylation-related genes based on TCGA transcriptomic and clinical data. We constructed a risk model with robust prognostic stratification capability and revealed significant differences among molecular subtypes as well as high- and low-risk groups in OS, PFS, and tumor microenvironment characteristics. These findings suggest that aberrations in sialylation-related molecules may have potential as biomarkers for the prognostic evaluation and prediction of the therapeutic response in BCa. On this basis, we further identified ST3GAL6 as a key molecule and demonstrated that it is not only highly expressed in tumor tissues and drug-resistant cells but also participates in the malignant progression of BCa by promoting cell proliferation, enhancing resistance to cisplatin and gemcitabine and activating the PI3K-AKT-mTOR pathway, indicating that it may serve as a potential therapeutic target with both biological significance and translational value. The reader IGF2BP3 acts upstream to drive the aberrant expression of ST3GAL6 by enhancing the stability of its mRNA, thereby further amplifying PI3K-AKT-mTOR signaling activity. Ultimately, this forms an “IGF2BP3–ST3GAL6–PI3K-AKT-mTOR” resistance-regulating axis that links epitranscriptomic dysregulation, glycosylation reprogramming, and activation of a canonical pro-survival pathway. However, our study has several limitations. The sialylation-related prognostic model established in this study was mainly derived from the TCGA database. Although it was preliminarily validated in the training and validation sets, it has not yet been confirmed in independent external cohorts or prospective clinical samples. Whether this model can be applied to different patient populations still requires more clinical evidence. Reduced IGF2BP3 expression decreased the stability of ST3GAL6 mRNA, suggesting that IGF2BP3 may help maintain ST3GAL6 expression. However, there is currently no direct evidence showing m6A modification on ST3GAL6 transcripts, and whether IGF2BP3 directly binds to ST3GAL6 mRNA remains unclear. Further studies are needed to verify this process. The functional validation of drug resistance in this study was mainly based on in vitro cell experiments. Although the results suggest that ST3GAL6 is associated with GC resistance, an in vivo GC treatment model is still needed to determine whether this regulatory axis affects chemotherapy response at the animal level. In future studies, we will use independent clinical cohorts, patient-derived organoids, PDX models, and in vivo GC treatment models to further evaluate this model and the related mechanisms. Overall, through multi-omics integration, bioinformatics modeling, drug-resistant cell models, and in vitro translational experiments, our study reveals a new molecular basis for GC resistance in BCa. It also provides a new theoretical foundation for biomarker discovery, mechanistic investigation, and targeted intervention in BCa based on genetic/epigenetic alterations, aberrant glycosylation, and signaling pathway remodeling.

## 5. Conclusions

We demonstrated that the reader IGF2BP3 acts upstream to drive the aberrant expression of ST3GAL6 by enhancing the stability of its mRNA, thereby further amplifying PI3K-AKT-mTOR signaling activity. Ultimately, this forms an “IGF2BP3–ST3GAL6–PI3K-AKT-mTOR” resistance-regulating axis that links post-transcriptional modification, glycosylation reprogramming, and activation of pro-survival signaling. Our findings not only provide a new theoretical basis for understanding the molecular mechanisms underlying gemcitabine–cisplatin resistance in BCa but also suggest that ST3GAL6 and IGF2BP3 may serve as promising biomarkers for prognostic evaluation and as potential therapeutic targets for reversing chemoresistance. Nevertheless, their clinical translational value still requires further validation in larger clinical cohorts and more comprehensive in vivo models of drug resistance.

## Figures and Tables

**Table 1:** OS analysis.

Gene	n_Low	n_High	Median OS (Low)	Median OS (High)	*χ* ^2^	*p*-Value	HR (95% CI)	*p* (HR)
*B3GALT2*	192	189	51.12	26.91	3.36	0.0666	1.33 (0.98~1.81)	0.0677
*SIGLEC6*	203	203	59.27	27.43	7.00	0.00816	1.49 (1.11~2.01)	0.00864
*SIGLEC10*	203	203	35.38	32.99	0.30	0.584	1.09 (0.81~1.46)	0.586
*ST3GAL5*	203	203	25.56	59.27	8.31	0.00395	0.64 (0.48~0.87)	0.00425
*ST3GAL6*	203	203	86.77	26.91	7.26	0.00704	1.51 (1.12~2.04)	0.00752

**Table 2:** PFS analysis.

Gene	n_Low	n_High	Median PFS (Low)	Median PFS (High)	*χ* ^2^	*p*-Value	HR (95% CI)	*p* (HR)
*B3GALT2*	192	190	54.70	21.85	3.68	0.055	1.35 (0.99~1.84)	0.056
*SIGLEC6*	203	201	43.17	29.47	0.08	0.772	1.04 (0.77~1.41)	0.773
*SIGLEC10*	203	200	25.23	32.56	0.12	0.729	0.95 (0.70~1.28)	0.728
*ST3GAL5*	203	203	20.70	51.42	9.79	0.00176	0.62 (0.46~0.84)	0.00194
*ST3GAL6*	203	203	55.16	21.82	6.79	0.00916	1.49 (1.10~2.02)	0.00962

**Table 3:** IGF2BP3 OS analysis.

Gene	n_Low	n_High	Median OS (Low)	Median OS (High)	*χ* ^2^	*p*-Value	HR (95% CI)	*p* (HR)
*IGF2BP3*	203	203	54.87	26.12	4.44	0.0352	1.38 (1.02~1.85)	0.036

## Data Availability

The data supporting the results of this study are available from the corresponding author upon reasonable request. Publicly available data analyzed in this study were obtained from The Cancer Genome Atlas (TCGA) database (https://www.cancer.gov/ccg/research/genome-sequencing/tcga, accessed on 26 October 2025). Some data related to clinical samples are not publicly available because of ethical and privacy restrictions.

## Supplementary Materials

The following supporting information can be downloaded at: https://www.mdpi.com/article/10.3390/cancers18111713/s1, File S1: WB Original Data Files. Figure S1: Prognostic model analysis. (A) Immune infiltration analysis of the C1, C2, and C3 subtypes. (B) Heatmap of gene expression patterns among different groups. (C) Predictive accuracy of the prognostic model for 1-, 3-, and 5-year survival (ALL: 1 years AUC = 0.646, 3 years AUC = 0.717, 5 years AUC = 0.713; TEST: 1 years AUC = 0.633, 3 years AUC = 0.663, 5 years AUC = 0.651; TRAIN: 1 years AUC = 0.658, 3 years AUC = 0.736, 5 years AUC = 0.735). * *p* < 0.05, ** *p* < 0.01, **** *p* < 0.0001. Figure S2: Effects of ST3GAL6 on BCa cell proliferation. (A) EdU assay validating the effect of ST3GAL6 on cell proliferation. (B) Flow cytometric analysis validating the effect of ST3GAL6 on cell cycle distribution. * *p* < 0.05, ** *p* < 0.01. Figure S3: Effects of ST3GAL6 on cisplatin and gemcitabine resistance in BCa. (A) Establishment of cisplatin- and gemcitabine-resistant BCa cell lines. (B) Patients with low ST3GAL6 expression show a more favorable outcome after cisplatin or gemcitabine treatment. (C,D) Establishment of stable cell lines with ST3GAL6 knockdown or overexpression. (E) Effects of ST3GAL6 overexpression on the IC50 values of cisplatin and gemcitabine in BCa cells. * *p* < 0.05, ** *p* < 0.01, *** *p* < 0.001. Figure S4: IGF2BP3 regulates BCa chemoresistance through ST3GAL6-mediated PI3K-AKT-mTOR signaling. (A) Correlation analysis between IGF2BP1 and ST3GAL6. (B) Immunohistochemical analysis of differential IGF2BP3 expression in BCa tissues and adjacent normal tissues. (C) Patients with low IGF2BP3 expression show a more favorable outcome after cisplatin or gemcitabine treatment. (D–F) IGF2BP3 is highly enriched in the PI3K-AKT-mTOR pathway across multiple tumor types. (G) ST3GAL6 overexpression reverses the suppression of PI3K-AKT-mTOR pathway activity caused by IGF2BP3 depletion. Table S1: Clinical information of the patients from whom the tissue specimens were obtained. Table S2: Sialylation-related gene set. Table S3: Genes included in the prognostic model and their model coefficients. Table S4: ST3GAL6 primer sequences, sh-ST3GAL6 sequences, and sh-IGF2BP3 sequences. Table S5: Antibody manufacturers and catalog numbers. Table S6: The drug concentrations used for cell treatment.

## Abbreviations

The following abbreviations are used in this manuscript:

BCa	bladder cancer
GC	cisplatin and gemcitabine
ROC	receiver operating characteristic
MIBC	muscle-invasive bladder cancer
m6A	N6-methyladenosine
NMF	non-negative matrix factorization
NMIBC	non-muscle-invasive bladder cancer
OS	overall survival
PFS	progression-free survival
AUC	area under the receiver operating characteristic curve
