# Supplementary material for: Abnormal Sialylation Promotes Chemotherapy Resistance in Bladder Cancer via the PI3K-AKT-mTOR Signaling Pathway
Source: Cancers (Basel). 2026 May 24;18(11):1713. doi: 10.3390/cancers18111713 (PMC13255594; doi:10.3390/cancers18111713)
Supplement: Supplementary file 1 [file cancers-18-01713-s001.zip › cancers-4267108-supplementary figure.pdf]

Supplementary Materials:

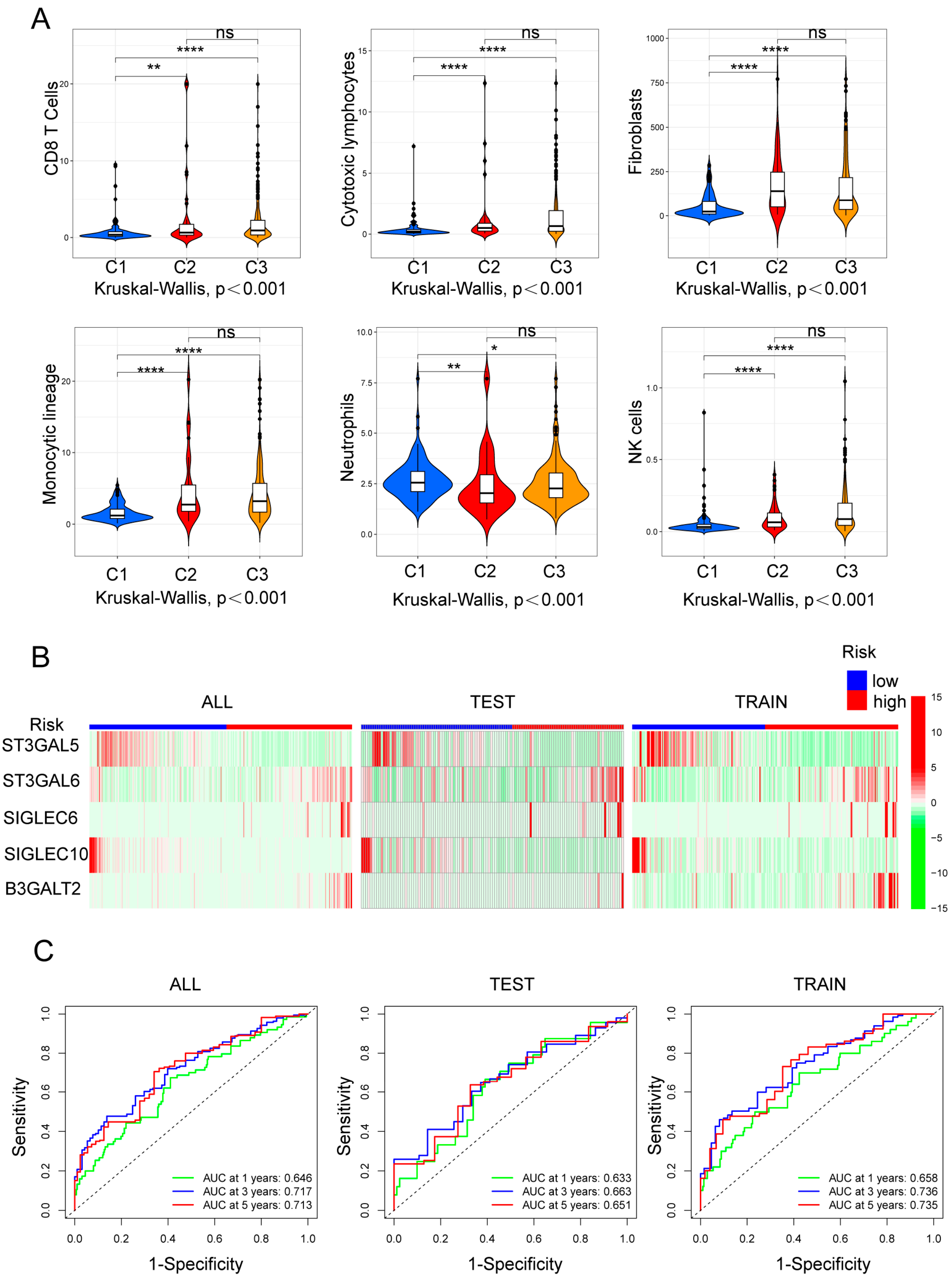

A
CD8 T Cells
Cytotoxic lymphocytes
Fibroblasts
Monocytic lineage
Neutrophils
NK cells
C1
C2
C3
ns
****
**
*
Kruskal-Wallis, p<0.001
B
Risk
low
high
ALL
TEST
TRAIN
ST3GAL5
ST3GAL6
SIGLEC6
SIGLEC10
B3GALT2
C
Sensitivity
1-Specificity
AUC at 1 years: 0.646
AUC at 3 years: 0.717
AUC at 5 years: 0.713
AUC at 1 years: 0.633
AUC at 3 years: 0.663
AUC at 5 years: 0.651
AUC at 1 years: 0.658
AUC at 3 years: 0.736
AUC at 5 years: 0.735

Supplementary Figure S1. Prognostic model analysis. (A) Immune infiltration analysis of the C1, C2, and C3 subtypes. (B) Heatmap of gene expression patterns among different groups. (C) Predictive accuracy of the prognostic model for 1-, 3-, and 5-year survival (ALL: 1 years AUC = 0.646, 3 years AUC = 0.717, 5 years AUC = 0.713; TEST: 1 years AUC = 0.633, 3 years AUC = 0.663, 5 years AUC = 0.651; TRAIN: 1 years AUC = 0.658, 3 years AUC = 0.736, 5 years AUC = 0.735). * $p < 0.05$, ** $p < 0.01$, **** $p < 0.0001$.

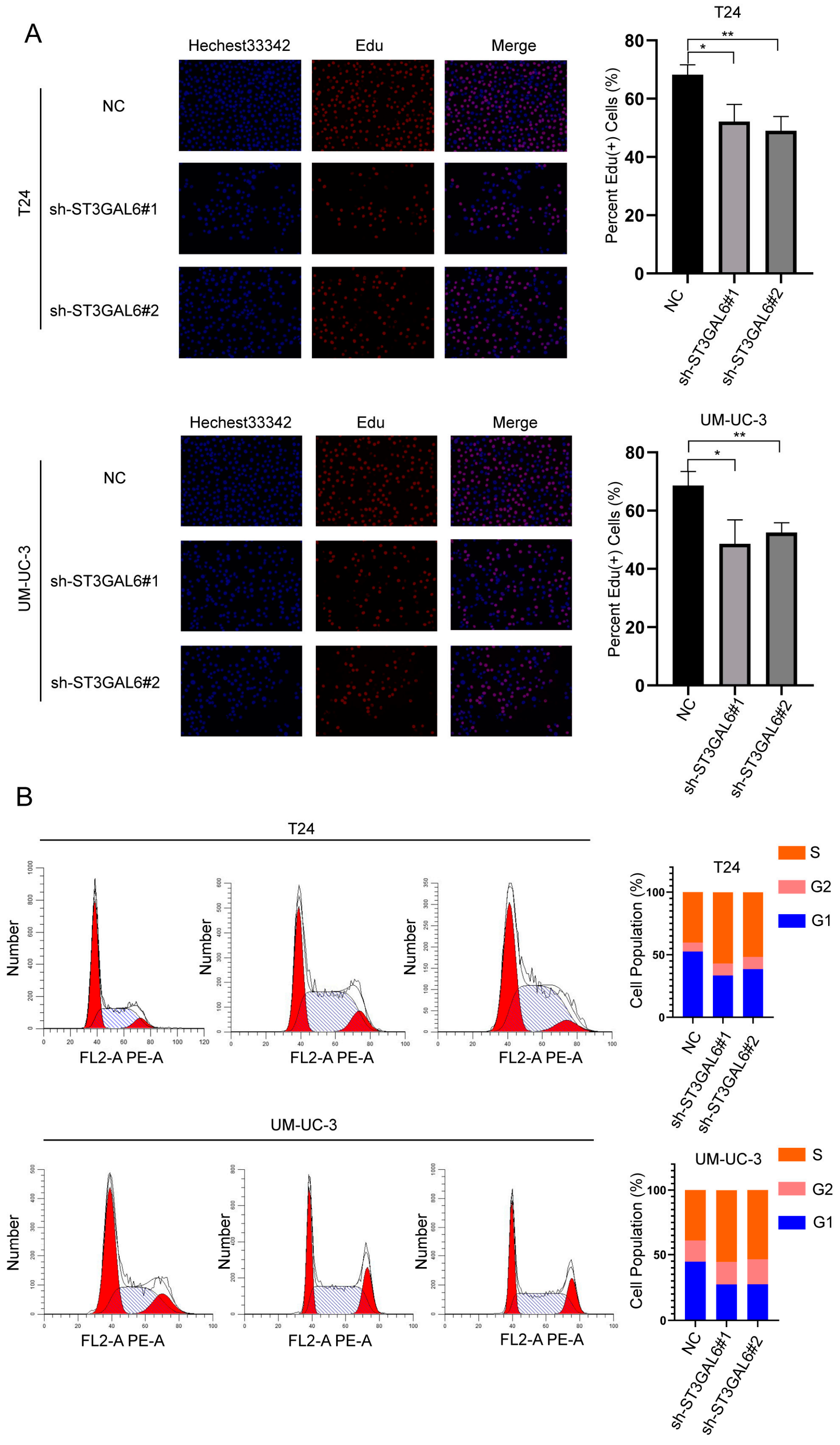

A
Hechest33342
Edu
Merge
T24
NC
sh-ST3GAL6#1
sh-ST3GAL6#2
Percent Edu(+) Cells (%)
UM-UC-3
B
Number
FL2-A PE-A
Cell Population (%)
S
G2
G1

Supplementary Figure S2. Effects of ST3GAL6 on BCa cell proliferation. (A) EdU assay validating the effect of ST3GAL6 on cell proliferation. (B) Flow cytometric analysis validating the effect of ST3GAL6 on cell cycle distribution. * $p < 0.05$, ** $p < 0.01$.

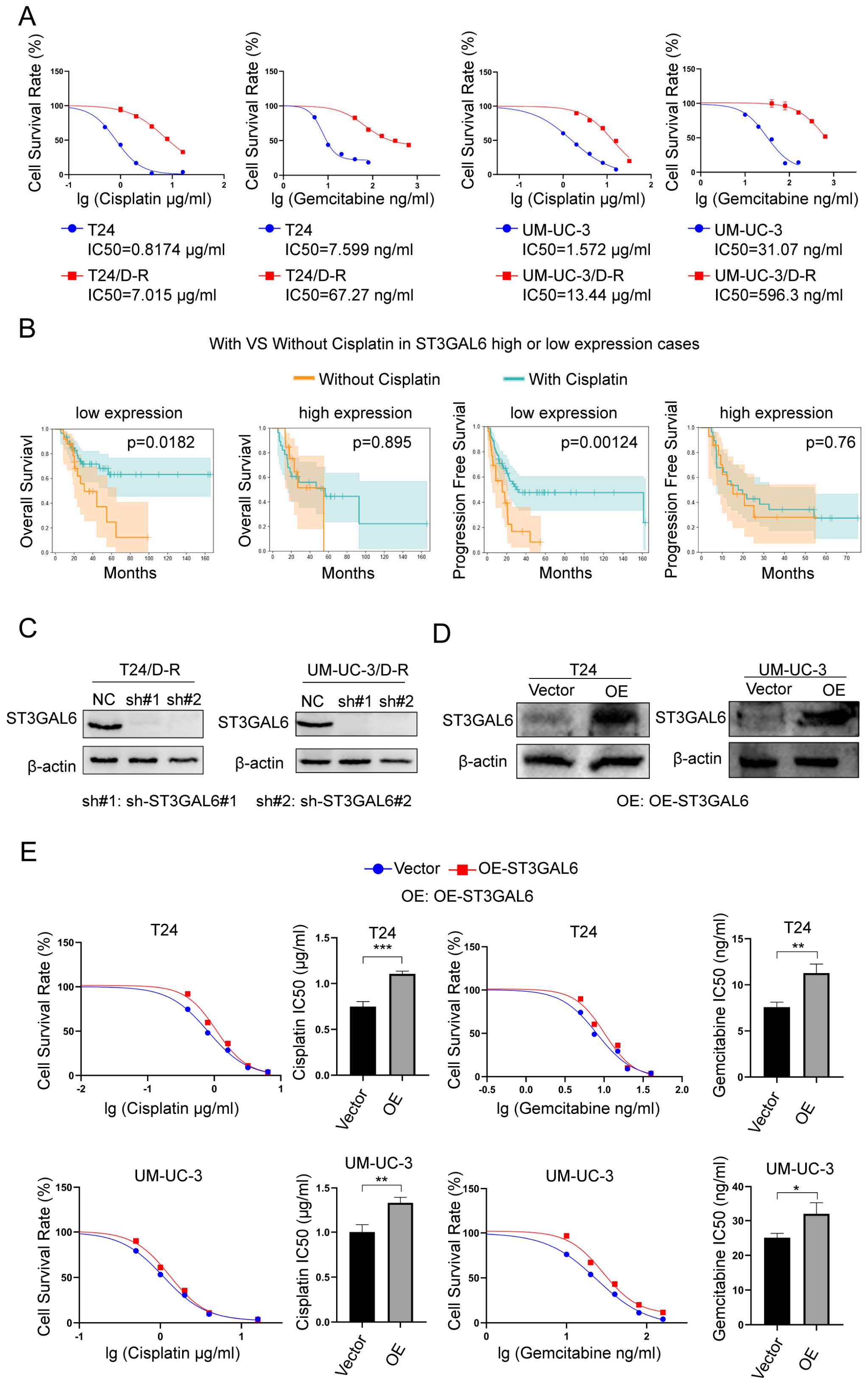
A
Cell Survival Rate (%)
lg (Cisplatin μg/ml)
lg (Gemcitabine ng/ml)
T24
IC50=0.8174 μg/ml
T24/D-R
IC50=7.015 μg/ml
T24
IC50=7.599 ng/ml
T24/D-R
IC50=67.27 ng/ml
UM-UC-3
IC50=1.572 μg/ml
UM-UC-3/D-R
IC50=13.44 μg/ml
UM-UC-3
IC50=31.07 ng/ml
UM-UC-3/D-R
IC50=596.3 ng/ml
B
With VS Without Cisplatin in ST3GAL6 high or low expression cases
Without Cisplatin
With Cisplatin
low expression
p=0.0182
high expression
p=0.895
low expression
p=0.00124
high expression
p=0.76
Overall Surviavl
Progression Free Survial
Months
C
T24/D-R
UM-UC-3/D-R
NC sh#1 sh#2
ST3GAL6
β-actin
sh#1: sh-ST3GAL6#1
sh#2: sh-ST3GAL6#2
D
T24
UM-UC-3
Vector OE
ST3GAL6
β-actin
OE: OE-ST3GAL6
E
Vector
OE-ST3GAL6
OE: OE-ST3GAL6
T24
UM-UC-3
Cell Survival Rate (%)
lg (Cisplatin μg/ml)
lg (Gemcitabine ng/ml)
Cisplatin IC50 (μg/ml)
Gemcitabine IC50 (ng/ml)
***
**
*
Vector
OE

Supplementary Figure S3. Effects of ST3GAL6 on cisplatin and gemcitabine resistance in BCa. (A) Establishment of cisplatin- and gemcitabine-resistant BCa cell lines. (B) Patients with low ST3GAL6 expression show a more favorable outcome after cisplatin or gemcitabine treatment. (C,D) Establishment of stable cell lines with ST3GAL6 knockdown or overexpression. (E) Effects of ST3GAL6 overexpression on the IC50 values of cisplatin and gemcitabine in BCa cells. * $p < 0.05$, ** $p < 0.01$, *** $p < 0.001$.

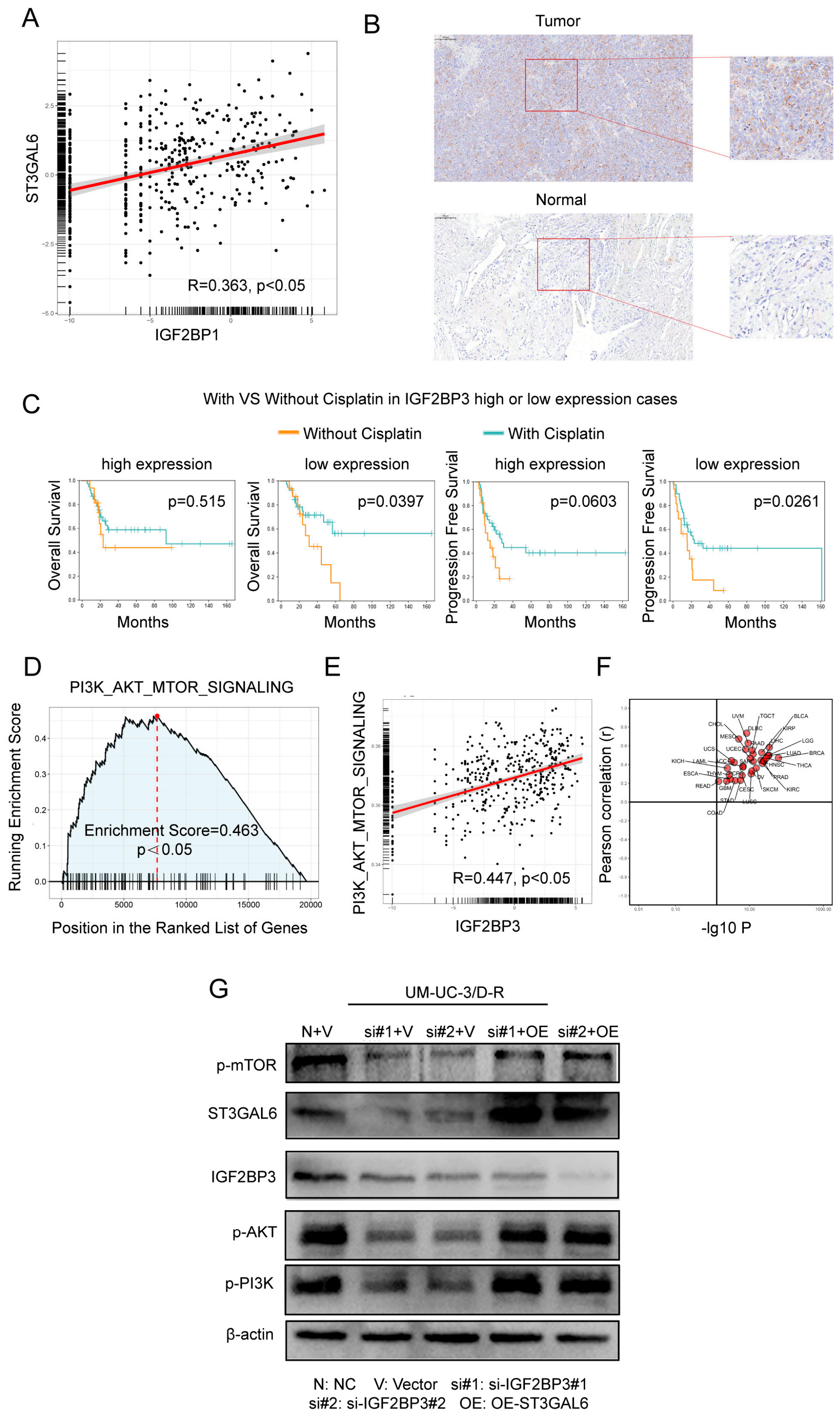
A
ST3GAL6
IGF2BP1
R=0.363, p<0.05
B
Tumor
Normal
C
With VS Without Cisplatin in IGF2BP3 high or low expression cases
Without Cisplatin
With Cisplatin
high expression
low expression
high expression
low expression
Overall Surviavl
Overall Surviavl
Progression Free Survial
Progression Free Survial
p=0.515
p=0.0397
p=0.0603
p=0.0261
Months
Months
Months
Months
D
PI3K_AKT_MTOR_SIGNALING
Running Enrichment Score
Enrichment Score=0.463
p<0.05
Position in the Ranked List of Genes
E
PI3K_AKT_MTOR_SIGNALING
R=0.447, p<0.05
IGF2BP3
F
Pearson correlation (r)
-lg10 P
G
UM-UC-3/D-R
N+V
si#1+V
si#2+V
si#1+OE
si#2+OE
p-mTOR
ST3GAL6
IGF2BP3
p-AKT
p-PI3K
β-actin
N: NC V: Vector si#1: si-IGF2BP3#1
si#2: si-IGF2BP3#2 OE: OE-ST3GAL6

Supplementary Figure S4. IGF2BP3 regulates BCa chemoresistance through ST3GAL6-mediated PI3K-AKT-mTOR signaling. (A) Correlation analysis between IGF2BP1 and ST3GAL6. (B) Immunohistochemical analysis of differential IGF2BP3 expression in BCa tissues and adjacent normal tissues. (C) Patients with low IGF2BP3 expression show a more favorable outcome after cisplatin or gemcitabine treatment. (D–F) IGF2BP3 is highly enriched in the PI3K-AKT-mTOR pathway across multiple tumor types. (G) ST3GAL6 overexpression reverses the suppression of PI3K-AKT-mTOR pathway activity caused by IGF2BP3 depletion.
