# Supplementary material for: Abnormal Sialylation Promotes Chemotherapy Resistance in Bladder Cancer via the PI3K-AKT-mTOR Signaling Pathway
Source: Cancers (Basel). 2026 May 24;18(11):1713. doi: 10.3390/cancers18111713 (PMC13255594; doi:10.3390/cancers18111713)
Supplement: Supplementary file 1 [file cancers-18-01713-s001.zip › cancers-4267108-supplementary tables.pdf]

Supplementary Materials:

Table S1. Clinical information of the patients from whom the tissue specimens were obtained.

Patient No.	Age	Sex	Muscle invasion status	Histological grade	T stage	Sample type
1	71	Male	MIBC	High	T2	Tumor and Adjacent
2	63	Female	MIBC	High	T3	Tumor and Adjacent
3	52	Male	MIBC	High	T4	Tumor and Adjacent
4	57	Male	MIBC	High	T3	Tumor and Adjacent
5	62	Male	MIBC	High	T3	Tumor and Adjacent

Table S2. Sialylation-related gene set.

sialylation-related genes
ST3GAL1,ST3GAL2,ST3GAL3,ST3GAL4,ST3GAL5,ST3GAL6,ST6GAL1,ST6GAL2,ST6GALNAC1,ST6GALNAC2,ST6GALNAC3,ST6GALNAC4,ST6GALNAC5,ST6GALNAC6,ST8SIA1,ST8SIA2,ST8SIA3,ST8SIA4,ST8SIA5,ST8SIA6,GNE,NANS,NANP,CMAS,SLC35A1,NEU1,NEU2,NEU3,NEU4,SIGLEC1,SIGLEC2,SIGLEC3,SIGLEC5,SIGLEC6,SIGLEC7,SIGLEC8,SIGLEC9,SIGLEC10,SIGLEC11,SIGLEC14,SIGLEC15,MAG,SELP,SELE,SELL,NPL,RENBP,B4GALT1,B4GALT2,B4GALT3,B4GALT4,B4GALT5,B4GALT6,B3GALT1,B3GALT2,B3GALT3,B3GALT4,B3GALT5,B3GALT6,GCNT1,GCNT2,GCNT3,GCNT4,FUT3,FUT5,FUT6,FUT7

Table S3. Genes included in the prognostic model and their model coefficients.

Genes	coef
ST3GAL5	-0.0305538296545316
ST3GAL6	0.463286295527345
SIGLEC6	0.071225797544082
SIGLEC10	-0.201880002683839
B3GALT2	1.19941075466793

.

Table S4. ST3GAL6 primer sequences, sh-ST3GAL6 sequences, and sh-IGF2BP3 sequences.

DesignationGenes	Genes	Sequences (5–3′)	Organism
Primer	ST3GAL6	F：ATTGCCATCACATTGGCGTTT	Homo sapiens
		R：GCAAAGGACTCTTGAGGTCAG	
	β-actin	F：CCTTCCTGGGCATGGAGTC	
		R：TGATCTTCATTGTGCTGGGTG	
sh-RNA	sh-ST3GAL6#1	CCGGTCCTCTATTATGTACTG-CATTCTCGAGAATGCAGTAC ATAATAGAGGATTTTTG	Homo sapiens
	sh-ST3GAL6#2	CCGGGATGAGAACATCAGCGGAA-TACTCGAGTATTCCGCT GATGTTCTCATCTTTTTG	
siRNA	si-IGF2BP3#1	Sense:GCAAAGGAUUCGGAAACU-UTT	Homo sapiens
		Antisense:AA-GUUUCCGAAUCCUUUGCTT	
	si-IGF2BP3#2	Sense:GGUGAAACUUGAA-GCUCAUTT	
		Antisense:AUGAUUCAAGUUUCAC-CTT	

.

Table S5. Antibody manufacturers and catalog numbers.

antibody	manufacturer	Cat. No.
ST3GAL6	Proteintech Group, Inc. (Wuhan, China)	13154-1-AP
IGF2BP3	Proteintech Group, Inc. (Wuhan, China)	14642-1-AP
PI3 Kinase (PI3K)	Abmart	T40064S
Phospho-PI3-kinase (P-PI3K)	Abmart	T40065S
Phospho-Akt (P-AKT)	Abmart	T40067S
AKT	Proteintech Group, Inc. (Wuhan, China)	10176-2-AP
mTOR	Abmart	T55306
Phospho-mTOR (P-mTOR)	Abmart	T56571S
β -actin	Proteintech Group, Inc. (Wuhan, China)	66009-1-Ig
AffiniPure Goat Anti-Rabbit IgG (H+L)	BOSTER	BA1039
AffiniPure Goat Anti-Mouse IgG (H+L)	BOSTER	BA1038

Table S6. The drug concentrations used for cell treatment.

antibody	manufacturer	Cat. No.
T24	Cisplatin	0、0.25、0.5、1、2、4μg/mL
T24/D-R	Cisplatin	0、2、8、16、32、64μg/mL
UM-UC-3	Cisplatin	0、0.25、0.5、1、2、4μg/mL
UM-UC-3/D-R	Cisplatin	0、2、8、16、32、64μg/mL
T24	Gemcitabine	0、5、10、20、40、80ng/mL
T24/D-R	Gemcitabine	0、20、80、100、200、300ng/mL
UM-UC-3	Gemcitabine	0、10、20、40、80、160ng/mL
UM-UC-3/D-R	Gemcitabine	0、300、600、800、1000、1500ng/mL
T24	Cisplatin	0、0.25、0.5、1、2、4μg/mL
T24/D-R	Cisplatin	0、2、8、16、32、64μg/mL
