# Supplementary material for: Abnormal Sialylation Promotes Chemotherapy Resistance in Bladder Cancer via the PI3K-AKT-mTOR Signaling Pathway
Source: Cancers (Basel). 2026 May 24;18(11):1713. doi: 10.3390/cancers18111713 (PMC13255594; doi:10.3390/cancers18111713)

Figure 2D

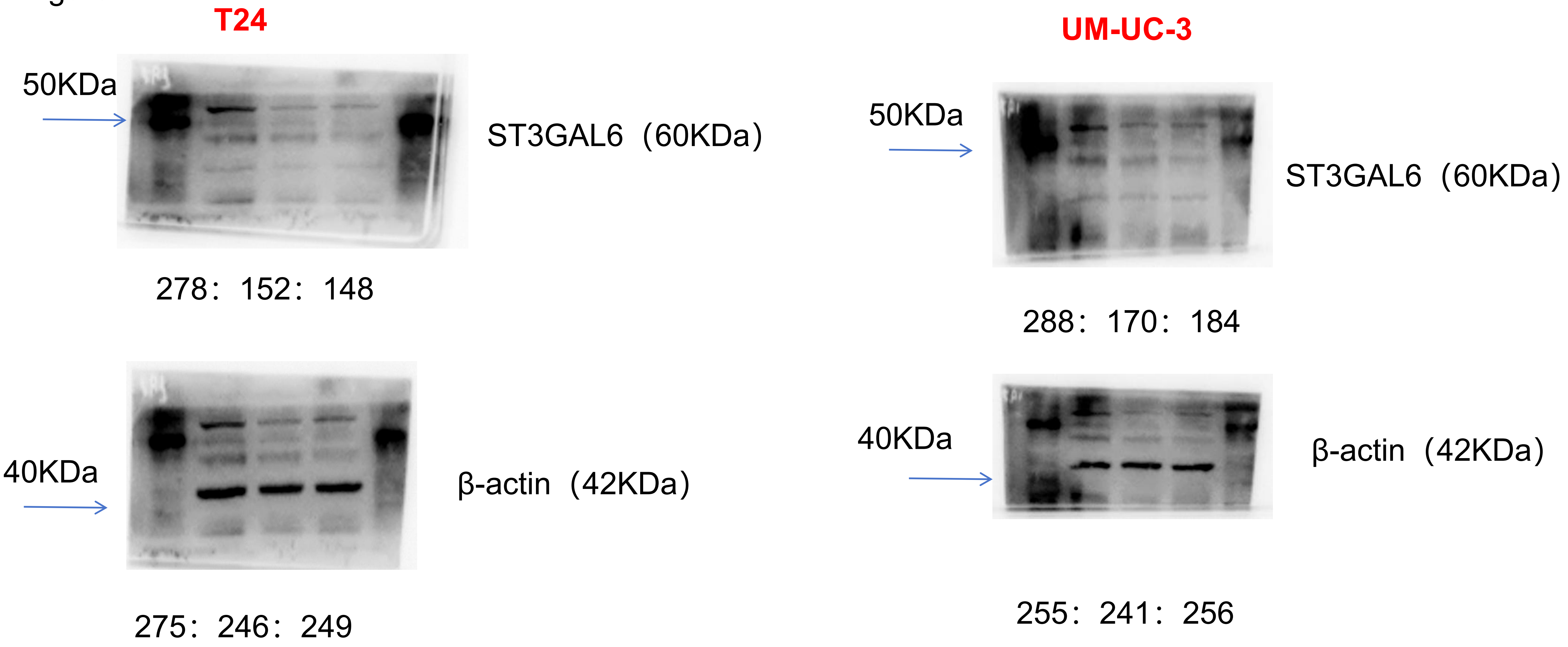
T24
50KDa
ST3GAL6 (60KDa)
278: 152: 148
40KDa
β-actin (42KDa)
275: 246: 249
UM-UC-3
50KDa
ST3GAL6 (60KDa)
288: 170: 184
40KDa
β-actin (42KDa)
255: 241: 256

Figure 4D

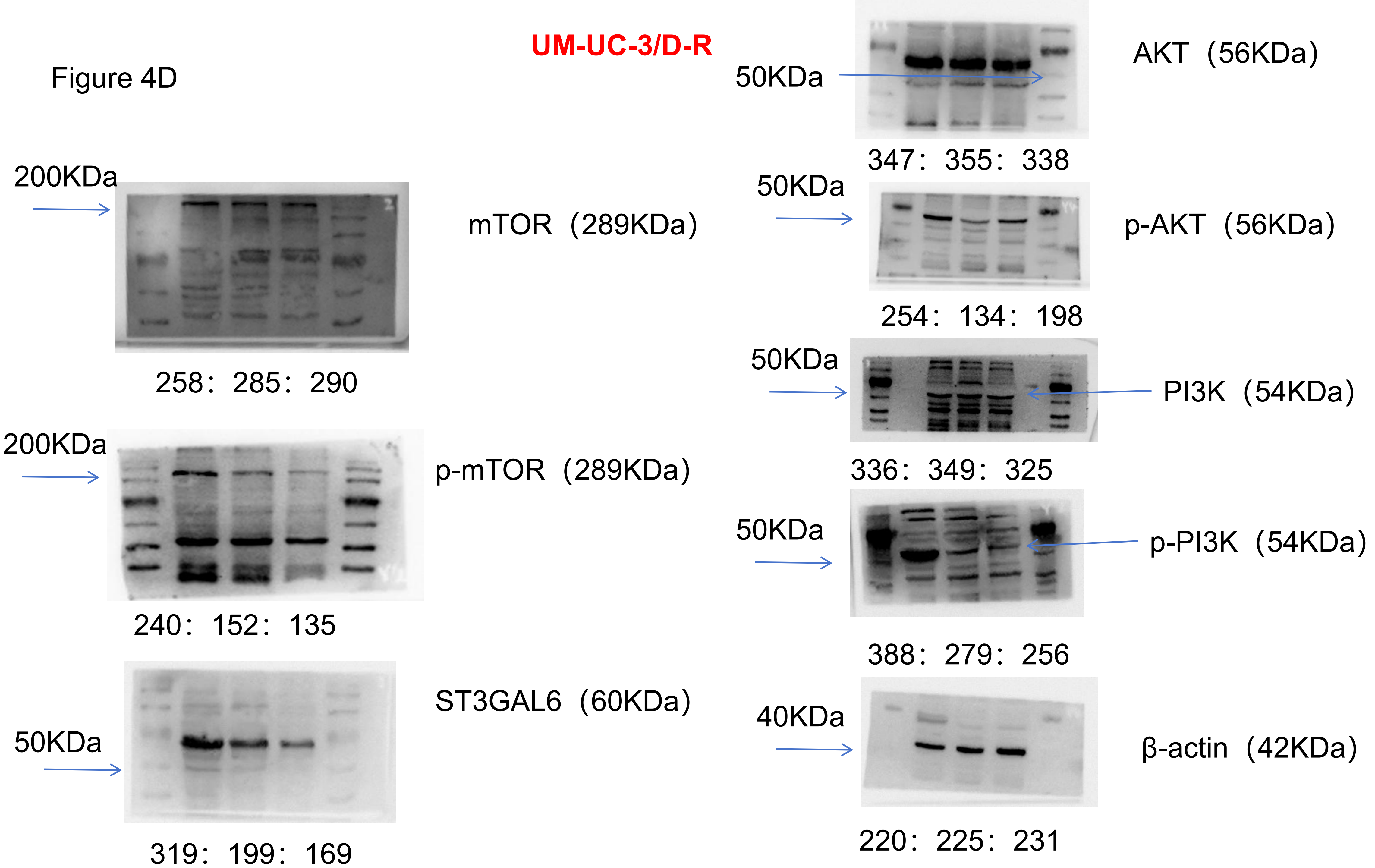
UM-UC-3/D-R
200KDa
mTOR（289KDa）
258：285：290
200KDa
p-mTOR（289KDa）
240：152：135
50KDa
ST3GAL6（60KDa）
319：199：169
50KDa
AKT（56KDa）
347：355：338
50KDa
p-AKT（56KDa）
254：134：198
50KDa
PI3K（54KDa）
336：349：325
50KDa
p-PI3K（54KDa）
388：279：256
40KDa
β-actin（42KDa）
220：225：231

Figure 4D

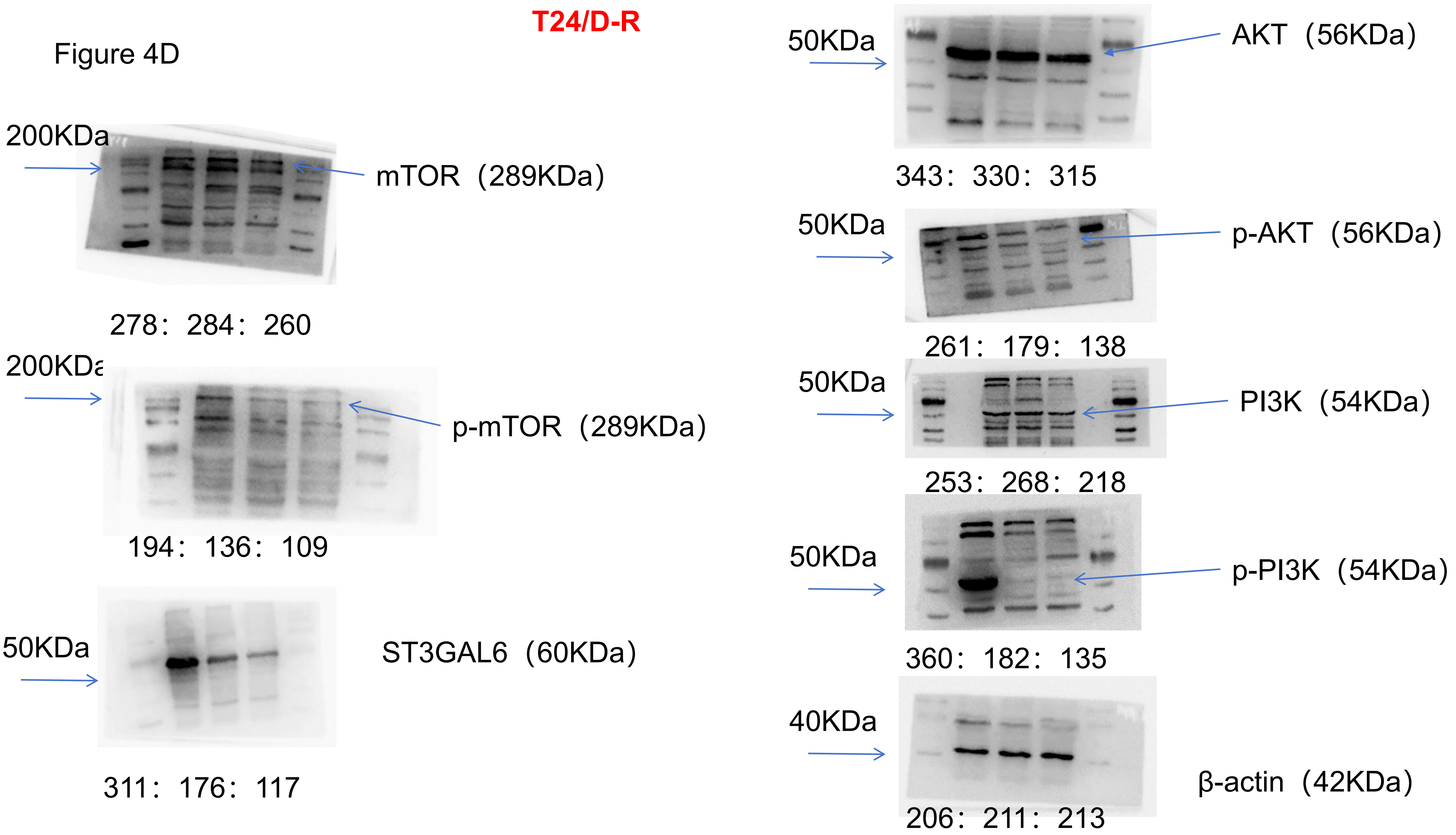
T24/D-R
200KDa
mTOR（289KDa)
278：284：260
200KDa
p-mTOR（289KDa)
194：136：109
50KDa
ST3GAL6（60KDa)
311：176：117
50KDa
AKT（56KDa)
343：330：315
50KDa
p-AKT（56KDa)
261：179：138
50KDa
PI3K（54KDa)
253：268：218
50KDa
p-PI3K（54KDa)
360：182：135
40KDa
β-actin（42KDa)
206：211：213

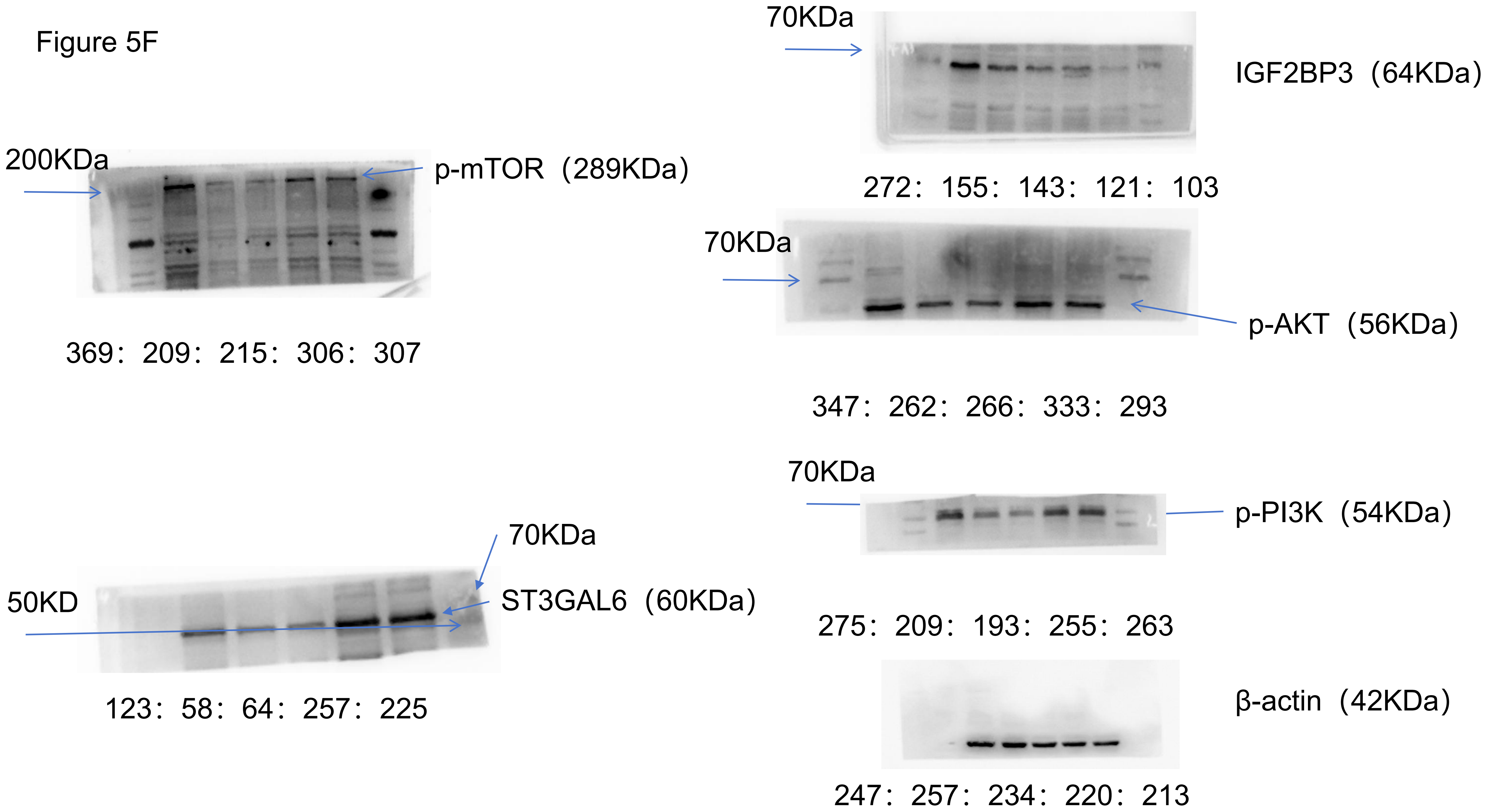
Figure 5F
70KDa
IGF2BP3 (64KDa)
272: 155: 143: 121: 103
200KDa
p-mTOR (289KDa)
369: 209: 215: 306: 307
70KDa
p-AKT (56KDa)
347: 262: 266: 333: 293
70KDa
p-PI3K (54KDa)
275: 209: 193: 255: 263
70KDa
50KD
ST3GAL6 (60KDa)
123: 58: 64: 257: 225
β-actin (42KDa)
247: 257: 234: 220: 213

Supplementary S3C

T24/D-R

70KDa

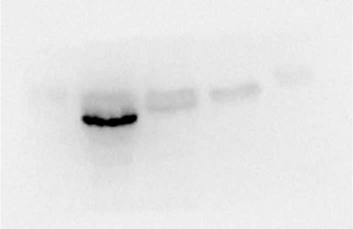

ST3GAL6（60KDa)

190： 74： 52

70KDa

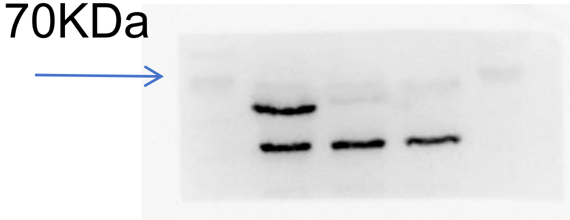

β-actin（42KDa)

162： 161： 129

UM-UC-3/D-R

70KDa

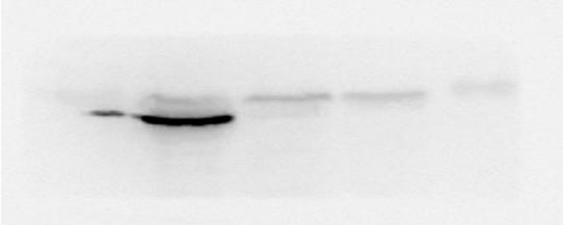

ST3GAL6（60KDa)

214： 96： 67

70KDa

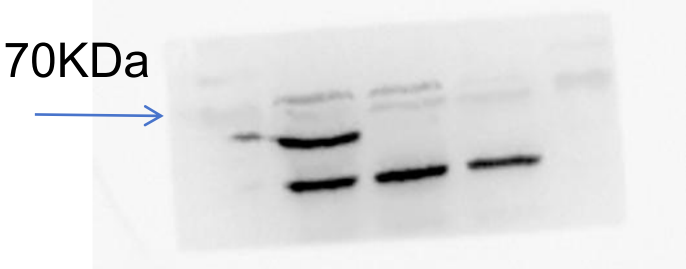

β-actin（42KDa)

178： 188： 120

Supplementary S3D

T24

70KDa

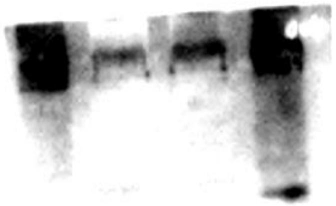

ST3GAL6（60KDa)

160：331

70KDa

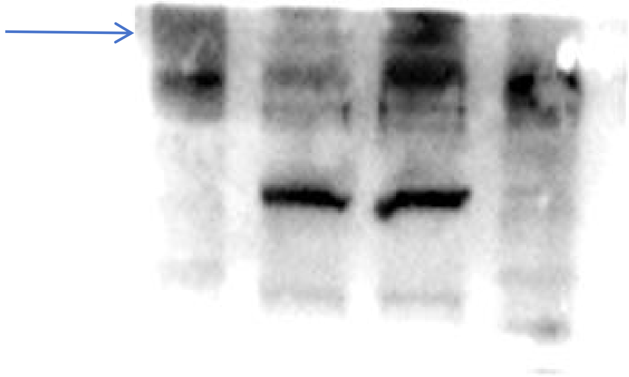

β-actin（42KDa)

184：201

UM-UC-3

70KDa

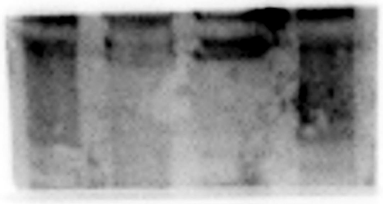

ST3GAL6（60KDa)

167：364

70KDa

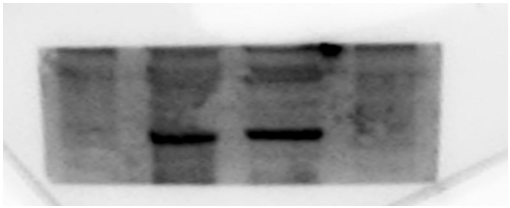

315：319

β-actin（42KDa)

Supplementary S4G

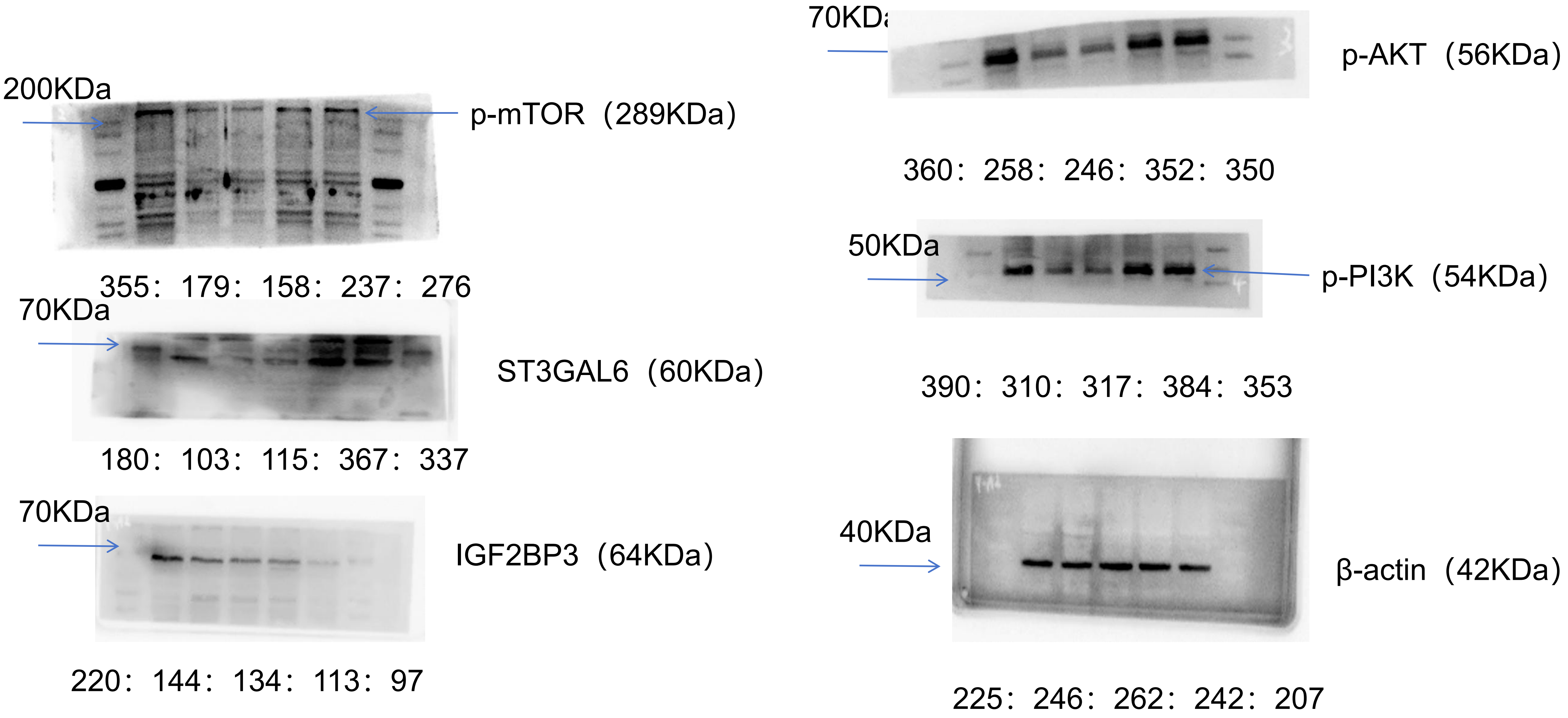

Supplement: Supplementary file 1 [file cancers-18-01713-s001.zip › File S1. WB Original Data Files.pdf]
